# The Role of PD-1/PD-L1 and IL-7 in Lymphocyte Dynamics and Sepsis Progression: A Biomarker Study in Critically Ill Patients

**DOI:** 10.3390/ijms252312612

**Published:** 2024-11-24

**Authors:** Oana Coman, Bianca-Liana Grigorescu, Adina Huțanu, Anca Bacârea, Anca Meda Văsieșiu, Raluca Ștefania Fodor, Marius Petrișor, Leonard Azamfirei

**Affiliations:** 1 Department of Simulation Applied in Medicine, University of Medicine, Pharmacy, Science and Technology “George Emil Palade”, 540142 Targu Mures, Romania; oana.coman@umfst.ro (O.C.); marius.petrisor@umfst.ro (M.P.); 2 Department of Anaesthesiology and Intensive Care, University of Medicine, Pharmacy, Science and Technology “George Emil Palade”, 540142 Targu Mures, Romania; raluca.fodor@umfst.ro (R.Ș.F.); leonard.azamfirei@umfst.ro (L.A.); 3 Department of Laboratory Medicine, University of Medicine, Pharmacy, Science and Technology “George Emil Palade”, 540142 Targu Mures, Romania; adina.hutanu@umfst.ro; 4 Centre for Advanced Medical and Pharmaceutical Research, Immunology, University of Medicine, Pharmacy, Science and Technology “George Emil Palade”, 540142 Targu Mures, Romania; 5 Department of Pathophysiology, University of Medicine, Pharmacy, Science and Technology “George Emil Palade”, 540142 Targu Mures, Romania; anca.bacarea@umfst.ro; 6 Department of Infectious Disease, University of Medicine, Pharmacy, Science and Technology “George Emil Palade”, 540142 Targu Mures, Romania; anca-meda.georgescu@umfst.ro

**Keywords:** sepsis, septic shock, apoptosis, lymphopenia, CD4+ lymphocytes, CD8+ lymphocytes, CD19+ lymphocytes, natural killer lymphocytes, PD-1/PD-L1 axis, IL-7

## Abstract

Sepsis pathophysiology involves a dysregulated immune response to infection, excessive inflammation, and immune paralysis. This study explores the relationships between cell death biomarkers (serum-soluble levels of programmed cell death protein 1 (PD-1), programmed death ligand 1 (PD-L1), and interleukin-7 (IL-7)) and the percentages of various lymphocyte subsets in relation to the severity and progression of sepsis. This prospective, observational study included 87 critically ill patients. We monitored parameters on days 1 (sepsis was diagnosed according to the Sepsis-3 Consensus) and 5. We established an IL-7 cutoff value of 1.94 pg/mL by comparing levels between a healthy control group and patients with sepsis (*p* < 0.0001). Lymphopenia was observed in all patients, with negative correlations between helper T lymphocytes and cytotoxic and B lymphocytes, and positive correlations involving cytotoxic lymphocytes across all groups. We found correlations between PD-1/PD-L1 and lymphocyte subsets. IL-7 showed a statistical correlation with PD-1 in non-survivors. Assessing lymphocyte levels shows potential as a biomarker for evaluating the progression of sepsis. Monitoring IL-7 levels could help assess survival, as low levels are associated with higher mortality risk. Monitoring IL-7 levels could help assess survival, as low levels are associated with higher mortality risk. Elevated PD-1/PD-L1 expression impairs costimulatory signalling, reducing T cell responses and lymphopenia, which increases the risk of nosocomial infections.

## 1. Introduction

Sepsis is a critical condition resulting from an imbalanced inflammatory response of the host to an infection, ultimately causing multiorgan failure. The inflammatory response contributes to the dysregulated reaction characteristic of sepsis, reflecting the host’s attempt to combat the progressing infection [1]. Despite advancements in medicine, sepsis remains the leading cause of mortality among critically ill patients, particularly elderly patients with multiple comorbidities [2,3]. In 2020, the World Health Organization (WHO) published its first global report on the epidemiology and burden of sepsis, highlighting that the estimated in-hospital mortality rate for sepsis patients treated in the intensive care unit (ICU) was over one-third, reaching 42% [4]. Timely assessment of the immune status in sepsis patients, combined with enhanced supportive therapy, can potentially reduce mortality rates [5]. However, it continues to be a significant challenge.

Inflammatory processes play a crucial role in the pathophysiology of organ dysfunction in critically ill patients. The susceptibility of these patients to secondary infections can be attributed to both an overstimulated innate immune response and a failed immune response, regardless of the initial insult [6].

The pathophysiology of sepsis is governed by the balance between proinflammatory and anti-inflammatory mechanisms. This balance is reflected in the continuous interaction of three pillars: lymphocyte subcategories, interleukin-7, and the modulatory component, the programmed cell death protein 1/programmed death ligand 1 (PD-1/PD-L1) axis. The clinical outcome of pathogen invasion hinges on the intricate interactions between the pathogen and immune cells, prompting a variety of defence mechanisms. Among these, cytokine production and cytotoxicity are the primary effector mechanisms in the acquired immune response [7,8]. During sepsis, both proinflammatory and anti-inflammatory cytokines are activated almost simultaneously, with the clinical picture primarily dominated by the proinflammatory phase. T helper cells (CD4+) play a significant role by secreting cytokines, while cytotoxic T lymphocytes (CD8+) and natural killer T CD3+ lymphocytes (NKT), the primary mediators of cytotoxicity, are involved in the pathogenesis of septic shock [7].

Interleukin 7 (IL-7) is a molecule known for its antiapoptotic properties and ability to induce significant proliferation of CD4+ and CD8+ T lymphocytes. IL-7 has been shown to prevent lymphocyte apoptosis, restore the functionality of CD4+ and CD8+ T cells, and improve survival rates in animal models of bacterial and fungal sepsis [9,10].

Immune checkpoints are immune-regulatory pathways indispensable for maintaining self-tolerance, preventing autoimmunity, and reducing collateral tissue damage. They act as brakes to modulate the adaptive immune response [11,12]. The interaction between programmed cell death protein 1 (PD-1) and its ligand, programmed death ligand 1 (PD-L1), is pivotal in dampening the activation signals triggered by the T cell receptor, also suppressing the immune response across various cell types [13,14].

Despite years of research in this field, the information available on diagnostic biomarkers for sepsis that can evaluate the inflammatory response to pathogens and reflect immune reactions is still limited.

This study aims to explore the relationships between cell death biomarkers (serum-soluble levels of PD-1, PD-L1, and IL-7) and the percentages of various lymphocyte subsets (CD4+ T helper lymphocytes, CD8+ T cytotoxic lymphocytes, natural killer T CD3+ lymphocytes, and CD19+ B lymphocytes) in relation to the severity and progression of sepsis.

## 2. Results

### 2.1. Population Analysis

A total of 87 patients were enrolled in this investigation: 31 female participants (35.63%) and 56 male participants (64.34%). The average age was 68 years, ranging from 33 to 90 years. The majority of patients (30) were between 71 and 80 years old, followed by 23 patients in the 61–70-year range. The smallest group consisted of just one patient in the 30–40-year range. The mean BMI was 28.9 ± 5.6, with a range between 15.60 and 49.40. Of the total, 63 patients (72.41%) had fatal outcomes, while 24 patients (27.59%) survived. The typical duration of stay in the intensive care unit was 14 days. Sepsis was prevalent in 57 patients (65.52%), and septic shock in 30 patients (34.48%).

In the study group, the prevalent underlying conditions and the infectious site varied as follows: cardiovascular disorders (83 patients, 83.91%), renal disease (58 patients, 69.88%), and respiratory disease (55 patients, 63.22%). As “Other” we included pathologies such as secondary anaemia, thrombocytopenia, chronic smoking, chronic alcohol intake, eschars, acid-base disorders, multiple organ dysfunction syndrome, hypovolemia, malnutrition, electrolyte disorders, etc., that can be found in Table S1.

Descriptive statistics for the entire study population and relevant demographic and clinical variables are provided in Table S2.

### 2.2. Lymphopenia

We studied the variation in lymphocytes for each subcategory of patients. We found that all patients presented lymphopenia on both studied days, with a statistically significant variation, an increase in % between day 1 and day 5, for sepsis and for survivor patients (Table 1).

### 2.3. Cutoff Value for IL-7

**Figure 1:**
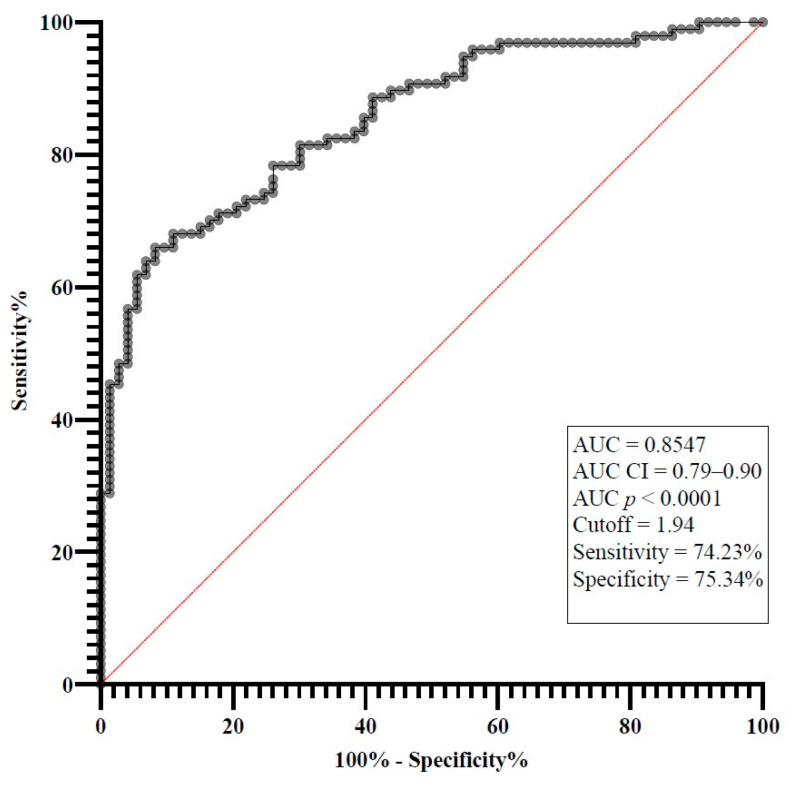
The standard ROC curve analysis of all patients and the control group.

The diagnostic accuracy of a biomarker is essential. Therefore, receiver operating characteristic (ROC) curve analysis was used to assess the diagnostic accuracy and determine the optimal cutpoint values for IL-7 levels between patients with sepsis/septic shock and control groups (Figure 1). According to the ROC curve of IL-7 analysis, the cutoff value for IL-7 was 1.94 pg/mL, with a sensitivity of 74.23% and a specificity of 75.34% for sepsis/septic shock, and the area under the curve (AUC) was 0.8547 (*p* < 0.0001), 95% CI 0.79–0.90.

Table 2 highlights the lowest and highest values of IL-7 in the four categories of patients. We counted the number of patients with a value of IL-7 above the identified cutoff value using the data found in Table S3.

### 2.4. Comparison of Variables for the Sepsis and Septic Shock Groups

We divided patients into sepsis and septic shock groups according to the Sepsis 3 Consensus criteria [1] and analysed the studied parameters on day 1 and day 5 after meeting the inclusion criteria. Comprehensive descriptive statistics for the study cohort, including demographic and clinical parameters, are presented in Table S4 for the sepsis group and Table S5 for the septic shock group.

**Figure 2:**
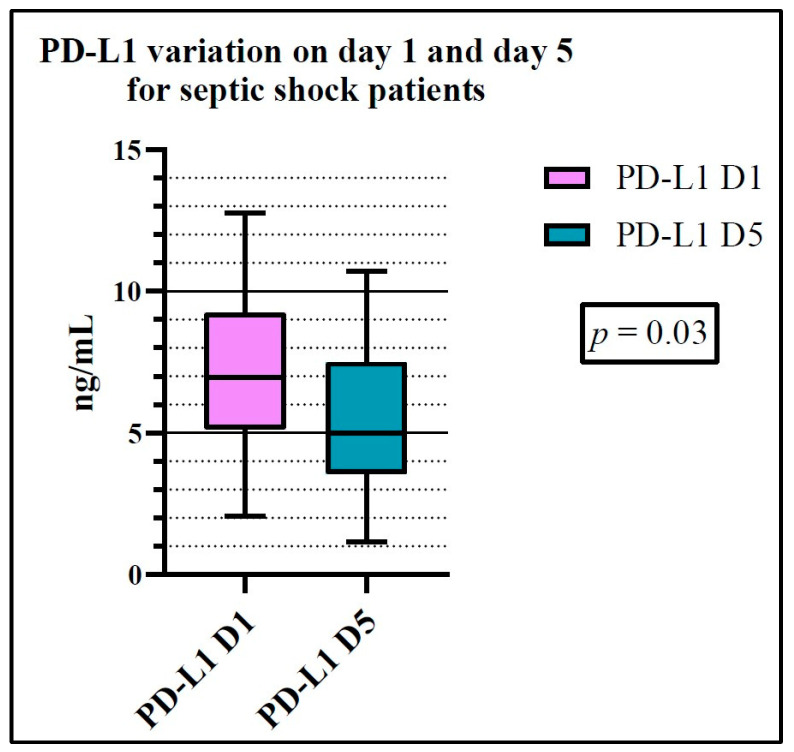
PD-L1 variation for septic shock patients between day 1 and day 5 (Wilcoxon test). D1: day 1, D5: day 5.

We observed a statistically significant variation for PD-L1 between days 1 and 5 (*p* = 0.0353) for septic shock patients (Figure 2). No other significant differences were observed (Table S6).

**Figure 3:**
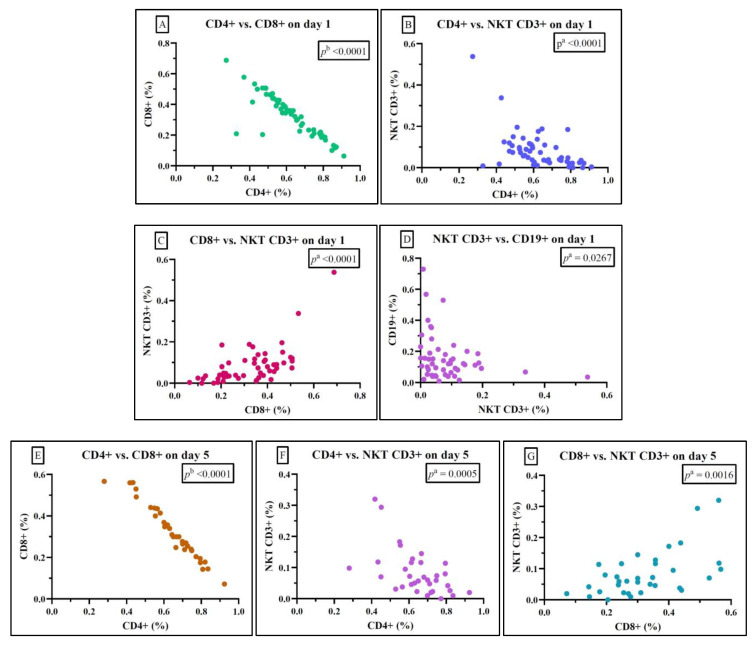
Statistically significant correlation between lymphocyte subtypes Th CD4+, Tc CD8+, and NKT on day 1 (**A**–**D**) and day 5 (**E**–**G**) in the sepsis group; ^a^ Spearman test, ^b^ Pearson test.

For the sepsis group, correlations between the subsets of lymphocytes pointed out statistically significant negative correlations between CD4+ and CD8+, and CD4+ and NKT CD3+ on both day 1 and day 5, and between NKT and CD19+ on day 1. We also observed a positive correlation between cytotoxic T cells (CD8+) and NKT on both study days (Figure 3 and Table S7).

**Figure 4:**
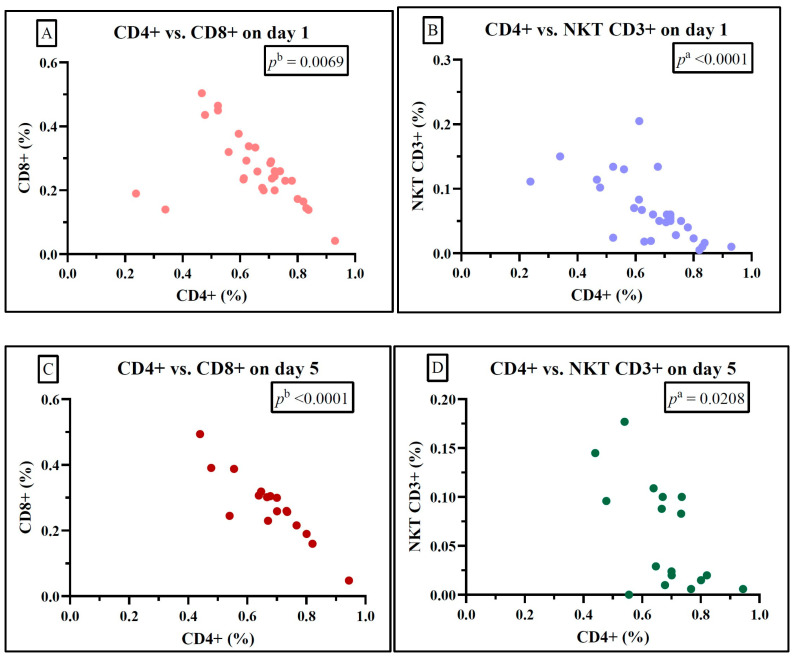
Statistically significant correlation between lymphocyte subtypes Th CD4+, Tc CD8+, and NKT on day 1 (**A**,**B**) and day 5 (**C**,**D**) in the septic shock group; ^a^ Spearman test, ^b^ Pearson test.

By correlating the lymphocyte subsets in the septic shock group, we observed statistically significant negative correlations only between CD4+ and CD8+ and between CD4+ and NKT on both day 1 and day 5 (Figure 4).

PD-1 was statistically positively correlated with PD-L1 on day 1 (r = 0.4566, *p* = 0.0190), and with IL-7 on day 5 (r = 0.5750, *p* = 0.0274). The rest of the correlations can be found in Table S8.

### 2.5. Comparison of Variables for the Survivor and Non-Survivor Groups

We divided the studied lot of patients into survivor and non-survivor groups and examined the studied parameters accordingly on day 1 and day 5 after meeting the inclusion criteria. Detailed descriptive statistics for the study population are available in Table S9 (survivor group) and Table S10 (non-survivor group).

**Figure 5:**
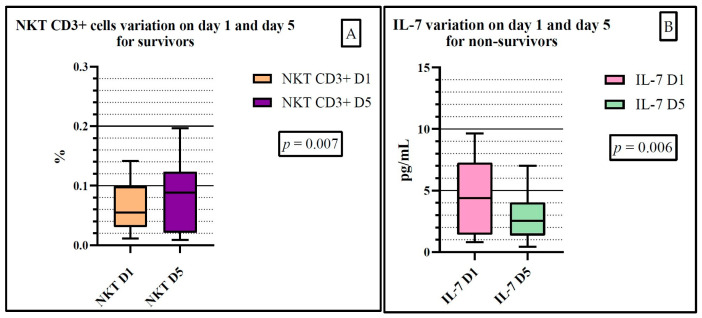
NKT lymphocyte variation for the survivor lot of patients between day 1 and day 5 (**A**). IL-7 variation for the non-survivor lot of patients between day 1 and day 5 (**B**). D1: day 1, D5: day 5.

We studied the variation in the parameters between the selected days. We observed a statistically significant variation for NKT lymphocytes (*p* = 0.076) in the survivor group, and a statistically significant variation for IL-7 (*p* = 0.069) in the non-survivor group (Figure 5). No other variation was significant (Table S11).

Correlating the subsets of lymphocytes, we observed statistically significant correlations between CD4+, CD8+, NKT, and CD19+ lymphocytes, as shown in Table 3 and Table 4.

For the survivor patients’ lot, statistically significant negative correlations were found between CD4+ and CD8+ and between CD8+ and CD19+ lymphocytes on both studied days, and between NKT and CD19+ on day 1, and between CD4+ and NKT on day 5. Statistically significant positive correlations were found between CD8+ and NKT on both studied days, and between CD4+ and CD19+ on day 5 (Table 3). The rest of the correlations can be found in Table S12.

For non-survivor patients, correlating the subsets of lymphocytes showed statistically significant negative correlations between CD4+ and CD8+, between CD4+ and NKT on both day 1 and day 5, and between NKT and CD19+ on day 1. Statistically significant positive correlations were found between CD8+ and NKT lymphocytes on day 1 and day 5 (Table 4). The rest of the correlations can be found in Table S13.

Correlating the lymphocyte subsets with the rest of the parameters, we observed a statistically significant positive correlation between T helper CD4+ lymphocytes and PD-1 on day 1 of study inclusion, whilst on day 5 we observed a negative correlation. For T cytotoxic CD8+ lymphocytes, we observed negative correlations on day 1 with PD-1 and PD-L1, and NKT correlated negatively with PD-1 on day 1. IL-7 correlated positively with the PD-1 component on day 5 (Table 5).

## 3. Discussion

Three consensus definitions of sepsis have guided physicians in understanding and managing this insidious disease. However, in 2016, these definitions were revised to address the significant limitations of previous versions, which included a simplistic model of the illness. The earlier model suggested that sepsis progresses from a systemic inflammatory response to a compensatory anti-inflammatory response syndrome. The Sepsis-3 definition has redefined the understanding of sepsis, encompassing the essence of the pathophysiology behind this disease. Nevertheless, much is still to understand [1,15].

In 2020, the WHO offered its first global report on sepsis, urging healthcare professionals to step up efforts to improve data about sepsis. The condition is linked to suboptimal quality of care, inadequate health infrastructure, poor infection prevention measures, delayed diagnosis, and inappropriate clinical management. Additionally, antimicrobial resistance complicates sepsis management in all settings, especially among high-risk populations, such as neonates and intensive care unit patients [4,16].

In 2017, a comprehensive global study identified 49 million sepsis cases and 11 million sepsis-related deaths; around 20% of worldwide annual deaths were related to sepsis. There were notable regional differences, with lower-middle-income countries experiencing the highest incidence and mortality rates. The estimated average hospital cost of treating sepsis was over USD 32,000 per patient; however, these figures primarily reflect data from high-income countries [2,17]. In the 2020 WHO report, the in-hospital mortality rate for sepsis patients receiving ICU treatment was estimated at 41.9%; for the European region, mortality reached 42.7%, with an incidence of 139/100,000 adults/year [4,18].

The development of organ dysfunction is the most critical clinical event during sepsis, as it is directly linked to mortality and morbidity. Despite the new definition by the Sepsis-3 Consensus, which emphasises sepsis as a “life-threatening organ dysfunction caused by a dysregulated host response to infection” [1], the mechanisms through which sepsis induces organ dysfunction remain incomplete. This knowledge gap is significant because mortality from sepsis remains very high, therapeutic options are limited, and nonspecific, and morbidity continues to be a substantial burden for patients after hospital discharge [19]. Nonetheless, the understanding of immunomodulatory mechanisms opens up opportunities for personalised immune therapies.

In our study, the three leading infectious sites were pulmonary, abdominal, and urinary tract, while the prevalent underlying conditions were cardiovascular, renal, respiratory, neurological, and diabetes. In a 2022 study, Prest et al. studied sepsis-related mortality trends based on the site of infection and found that pulmonary, abdominal, and genitourinary sepsis were the leading causes of mortality in the United States of America; demographical factors, such as age, sex, and race, played an important role [20]. In a 2022 study, Chen et al. examined the relationship between infection site and mortality in patients with cancer and sepsis or septic shock. They found that lung, urinary tract, unspecified site, and gastrointestinal infections were the most common, with gastrointestinal infections and pneumonia showing the highest mortality rates [21].

To develop new immunotherapy approaches for patients with sepsis and septic shock, it is essential to have a thorough understanding of the extensive changes occurring in the immune system. In recent years, there has been increasing emphasis on strategies aimed at effectively mitigating the immunosuppressive state in sepsis patients. A timely and accurate assessment of immune status is crucial for promptly identifying immune dysfunction in these patients and determining the optimal timing for administering immunomodulatory therapy [3,22].

Lymphocytes play a crucial role in both initiating and sustaining the body’s response to sepsis. Their significance lies in their ability to interact with the innate and adaptive immune systems, as well as their capacity to coordinate, amplify, and regulate the inflammatory response. Lymphocyte anergy or loss of their numbers can significantly impair the immune system [23].

Our study observed lymphopenia in all the studied categories of patients from day 1 of study inclusion compared to the standard laboratory values. On day 5, we observed an increase in the medians of the lymphocyte count, especially in the sepsis and survivor groups, without statistical significance. A statistically significant variation was observed in the percentage of total lymphocytes in the sepsis and survivor groups. In a study by Drewry et al. involving 335 patients hospitalised with bacterial or fungal sepsis, lymphopenia was observed in both survivors and non-survivors on the first day. However, by day 4, a notable difference emerged: Survivors had higher lymphocyte counts compared to non-survivors [24]. Another study, published by Hohlstein et al., assessed 105 ICU patients (52 of whom had sepsis) and found that survivors had significantly higher baseline counts of leukocytes, lymphocytes, and T cells. Notably, patients with a T cell count below 0.36 × 10⁹/L at admission had a significantly higher 100-day mortality rate (60%) compared to those with a count above the same value (20%) [25]. Two studies observed that a low lymphocyte count sustained for more than 3 days is a predictor of 28-day mortality [24,26], and one study found that lymphocyte percentage is a significant predictor of prognosis in patients with lung cancer [27], supporting our findings of persistent lymphopenia in the non-survivor group on day 5, together with the increased mortality rate of 72.41%. However, lymphocyte percentage has not yet been proven to be a reliable tool in sepsis evolution and prognosis.

A hallmark of sepsis is the significant depletion of lymphocytes due to apoptosis. Postmortem examinations of patients who succumbed to sepsis revealed a substantial loss of lymphocytes in the spleen, intestines, and other organs [28,29]. Lymphopenia involves the loss of various lymphocyte classes, including CD4+ and CD8+ T cells, B cells, and natural killer cells [28]. The T lymphocyte response becomes highly dysfunctional, and a state of T cell exhaustion rapidly develops following sepsis [30,31].

A reduction in total lymphocyte count and lymphocyte infiltration in pathological specimens is linked to poor overall survival [32]. IL-7 is an essential cytokine for the immune system, and deficiencies in IL-7 or its receptor can result in severely impaired development of immune cells. Studies have shown that IL-7 is essential for the development and maintenance of various immune cells. It aids lymphocyte development in the thymus, supports lymph node formation, and sustains activated T cells in secondary lymphoid organs. Increased IL-7 production enhances the survival of both naïve and memory T cells. IL-7 is also implicated in multiple stages of B-cell progenitor development, including commitment, survival, differentiation, and proliferation. Additionally, IL-7 is a critical cytokine that regulates the recruitment of leukocytes, such as neutrophils and monocytes [28,33,34,35].

Recent literature regarding the IL-7 cutoff value is scarce, especially concerning sepsis and septic shock. The value of IL-7 has been studied thoroughly in cancer patients, radiation-induced lymphopenia patients, and HIV patients [32,34,36]. Analysing the sepsis/septic shock groups, on day 1 of study inclusion, with the healthy control group, we found the cutoff value of IL-7 to be 1.94 pg/mL with an AUC of 0.8547 (*p* < 0.0001). Pathologic values in the aforementioned studied groups were considered those above the cutoff value of 1.94 pg/mL. We observed that on day 1, the median IL-7 levels in both the sepsis and septic shock groups were elevated, surpassing the identified cutoff value. Although a decrease by day 5 was not statistically significant, the median value remained elevated above the cutoff threshold.

In the survivor and non-survivor groups, we observed a similar result: an increased median value of IL-7, above the cutoff value, and a gradual decrease towards day 5, with a statistically significant variation for the non-survivor group. This is explained by the fact that IL-7 is a powerful antiapoptotic cytokine produced in various organs, including the thymus gland, liver, peripheral lymphoid organs, and in small amounts by dendritic cells. It plays an essential role in lymphocyte survival, development, and expansion [23]. The increased values of IL-7 above the cutoff threshold and maintained throughout the studied period, observed in our studied groups, serve as a means to restore depleted lymphocyte populations [37]. The statistically significant decrease in IL-7 in the non-survivor group is translated into lymphocyte fatigue and functional exhaustion, predisposing the patient to secondary infections and subsequent immunosuppression in individuals who survive the initial septic episode [1,23]. A study from 2023 by Leśnik et al. found a cutoff value for IL-7 on admission day for the septic shock group of 15.8 ng/l (15.8 pg/mL) and an AUC of 0.556 (*p* = 0.81), the values gradually decreasing on days 3 and 5 [9]. The cutoff value of 15.8 pg/mL is superimposed on the maximum value of Il-7 that we found in the septic shock group on day 5 (15.3 pg/mL).

In sepsis, immunosuppression involves various cell types and mechanisms, most notably the increased apoptosis of T and B cells, T cell exhaustion, and reduced expression of activating surface molecules. The apoptosis of immune cells is particularly pronounced in CD4+ T cells, CD8+ T cells, B cells, natural killer cells, and follicular dendritic cells [7,38].

For the sepsis group, we observed a statistically significant negative correlation between CD4+ and CD8+ and between CD4+ and NKT on both studied days, suggesting a decrease in the value of both helper and cytotoxic lymphocytes. We observed the same statistically significant negative correlation between CD4+ and CD8+ and between CD4+ and NKT in the septic shock group. Similar negative correlations were found in the survivor and non-survivor groups. The negative correlation between helper T lymphocytes and cytotoxic lymphocytes, irrespective of sepsis or septic shock, can be attributed to profound immunosuppression and heightened inflammation. The fluctuation in T lymphocytes is indispensable for the immune response against sepsis. Nevertheless, even if lymphocyte counts remain within the normal range, immunoparalysis can severely impair their function [23,39]. These findings are consistent with recent studies that have also reported a significant reduction in CD4+ cells among sepsis patients [5].

Known for their role in adaptive immunity, helper T lymphocytes activate B cells and cytotoxic T lymphocytes [39]. Our study observed a statistically significant negative correlation between NKT lymphocytes and CD19+ B lymphocytes, in the sepsis group, survivor and non-survivor groups, on day 1. This is sustained by how sepsis affects these lymphocyte subpopulations. B lymphocytes are central to the adaptive immune response, producing antigen-specific antibodies targeting pathogens. In sepsis, they become dysregulated and exhausted, rendering the cells of the adaptive immune system incapable of mounting an effective defence against infection. A recent study by Wang et al. found that the frequency and absolute number of regulatory B lymphocytes (Bregs) were significantly decreased in septic patients compared with healthy controls, and that low levels of Bregs were associated with an increased risk of non-survival in septic patients [40].

NKT lymphocytes are a unique subset of T cells, primarily acting in the body’s defence against infections. NKT cells are abundant in liver, spleen, and peripheral blood tissues. They respond rapidly to various pathogens and perform multiple roles, including cytotoxic activity, supporting antibody production, influencing Th1/Th2 differentiation, and linking innate and adaptive immune responses. In sepsis, NKT cells are important initiators of immune responses, where they activate and regulate downstream effector immune cells and trigger an inflammatory cytokine cascade, supporting pathogen clearance and immune response coordination. [41,42]. The role of NKT cells in regulating sepsis is complex. While they trigger an inflammatory cytokine cascade necessary for clearing pathogens, an excessive inflammatory response can negatively impact patient outcomes. The heightened inflammatory activity can lead to NKT cell apoptosis and immunosuppression, significantly contributing to sepsis mortality [41].

An interesting finding of our study is the statistically significant variation between day 1 and day 5 for NKT lymphocytes, an increase in the median value towards day 5 for the survivor group. Although not statistically significant, we observed a reversal of the medians in the septic shock and non-survivor groups, with a decrease towards day 5. In a 2015 study, Young et al. observed that a significant decrease in NKT lymphocytes due to apoptosis reduces IFN-gamma secretion, thus contributing to immunosuppression in sepsis non-survivors [43]. However, the mechanisms behind NKT cell apoptosis triggered by sepsis remain poorly understood. In 2023, Wu et al. found that NKT cell subsets were significantly reduced by up to 60% within the first two days following sepsis onset, primarily due to increased apoptosis [41]. In our study, the increased median value of NKT on the fifth day compared to the first day in the sepsis group places the subjects in the initial hyperinflammatory phase of sepsis; meanwhile, the reversed median values for the septic shock and non-survivor patients represent the immunosuppression manifested in the late stages of sepsis, together with functional exhaustion and anergy.

In our study, the cytotoxic answer can be observed by the statistically significant positive correlation between CD8+ T cytotoxic lymphocytes and NKT lymphocytes on day 1 and day 5 in the sepsis group, a correlation found in the survivor and non-survivor groups as well. Doganyigit et al. published a study stating that cytotoxic T lymphocytes are key components of the cytotoxic cell axis, playing an important role in defending the host by lysing cells infected with intracellular pathogens [8]. Another study published by Chen et al., investigating the immune status of CD8+ lymphocytes by examining the expression of granzyme A, granzyme B, and perforin, found that in septic shock non-survivors the activated CD8+ T lymphocytes were consumed, followed by a suppression of their immune function, but in septic shock survivors, there was a compensatory increase [7], suggesting that a rebalancing surge of cytotoxic players occurs during sepsis. These studies endorse our findings that CD8+ and NKT correlate positively in the sepsis group and in both survivors and non-survivors groups.

Immune checkpoint inhibitory receptors on immune cells trigger signalling pathways promoting immunosuppression. These molecules function as regulatory mechanisms, similar to brakes, moderating the adaptive immune response. PD-1, an immune checkpoint receptor, plays a pivotal role in regulating both the quantity and functional capacity of T cells [44]. PD-1 is expressed on T cells, B cells, antigen-presenting cells, and some non-lymphoid tissues. When ligands bind to PD-1 receptors on T cells, it leads to immune suppression by inhibiting T-cell activation and encouraging apoptosis. Additionally, PD-L1 is found in cardiac endothelial cells, placental tissues, and pancreatic islets, indicating its role in promoting immunological tolerance [45,46,47]. Soluble forms of PD-1 and PD-L1 can be produced by shedding the membrane-bound forms or as alternative splice variants. PD-1 has both a membrane-bound form and a soluble form, known as soluble PD-1 (sPD-1), which enters the bloodstream and acts similarly to a cytokine, helping to regulate the immune response. Recently, sPD-1 has gained attention as a crucial natural inhibitor that helps maintain the balance of the PD-1/PD-L1 signalling pathway. It has been linked to risk stratification and prognosis assessment [48,49]. Zhao et al. studied the soluble form of PD-L1, which regulates the PD-1/PD-L1 axis. During sepsis, serum levels of sPD-L1 are elevated, which may indicate the severity of the condition and predict clinical outcomes [50].

In non-survivors, we observed strong evidence of immunosuppression, indicated by a statistically significant positive correlation between CD4+ helper cells and PD-1 on day 1, which shifted to a statistically significant negative correlation by day 5 of the study. Similar correlations were found between CD4+ and PD-L1, although not statistically significant. Also, statistically significant negative correlations on day 1 between CD8+ cytotoxic T lymphocytes, PD-1, and PD-L1, together with a negative correlation between NKT and PD-1 on day 1, suggest an increased inflammatory response leading to immune suppression. These align with findings from Shao et al., who reported increased PD-L1 expression on immune cells in septic shock patients, which has been associated with higher mortality rates within the first 28 days of illness. The study also noted the activation of inhibitory immune checkpoints, metabolic disruptions, impaired effector cytokine production, reduced cell proliferation, and increased susceptibility to apoptotic cell death [13,51].

To enhance the profound immunosuppression brought by the expression of the PD-1/PD-L1 axis, we found a statistically significant positive correlation in the septic shock group on the first day of study between PD-1 and PD-L1. Despite not being statistically significant, a positive correlation between the two components was found on day 5 as well. In a previously published study [52], we found positive correlations between PD-L1 and the sequential organ failure assessment score, reflecting the presence of multiple organ dysfunction and an immunosuppressed status and increasing the risk of mortality in these patients. In a separate study, Zhao et al. discovered that non-survivors of sepsis had higher peripheral blood levels of soluble PD-1 and PD-L1, as well as increased PD-1 expression on CD4+ and CD8+ T cells and PD-L1 expression on monocytes, compared to survivors. Additionally, the levels of soluble PD-1 and PD-L1 were found to correlate with the severity of the disease [50]. Enhanced PD-1/PD-L1 axis expression increases mortality and the development of secondary infections [53,54].

The battle between cell survival and cell death mechanisms can be seen in the statistically significant positive correlation we found on day 5 in the septic shock and non-survivor groups between PD-1 and IL-7. Recent studies have shown that PD-1 expression levels on responding T lymphocytes decrease when the activated antigen is eliminated. If the antigen is not cleared, as seen in persistent infections and malignancies, PD-1 expression remains elevated [13], rendering T cells fatigued, dysfunctional, vulnerable to apoptosis, and unable to participate in an effective immune response [44,55]. Conversely, IL-7 enhances cell survival by stimulating the strong proliferation of CD4+ and CD8+ T lymphocytes, preventing lymphocyte apoptosis, restoring CD4+ and CD8+ T cell function, and ultimately improving survival [9].

This study has several limitations. First, we measured only the serum-soluble levels of PD-1 and PD-L1 without assessing the expression of membrane-bound PD-1/PD-L1. It is essential to highlight that detecting serum-soluble PD-1/PD-L1 is easier and more cost-effective than detecting the membrane-bound forms, which also speeds up disease diagnosis in clinical settings. Second, our study cannot affirm that there is a direct relationship between serum-soluble PD-1/PD-L1 and the variations in certain lymphocyte subsets. This can only be achieved by measuring the expression of membrane-bound PD-1/PD-L1 on lymphocytes. Nevertheless, the clinical use of serum-soluble PD-1/PD-L1 testing in sepsis shows valuable predictive capacity, aiding the physician in reaching the outcome. Third, the relatively small sample size made it difficult to draw definitive conclusions. Furthermore, as a single-centre study, there was potential bias in evaluating the pathology. In the future, the authors plan to expand the study group and further evaluate the examined parameters in a larger patient cohort.

## 4. Materials and Methods

### 4.1. Ethical Approval

This prospective observational single-centre study enrolled 87 sepsis patients admitted to the ICU of the County Emergency Clinical Hospital in Târgu Mureș, Romania, between July 2021 and March 2023.

The study was conducted in accordance with the Helsinki Declaration of 1975. Ethical approval for this study was obtained from the Ethics Committee of the University of Medicine and Pharmacy, Science, and Technology ‘George Emil Palade’ in Târgu Mureș (Mureș, Romania; approval no 1425/01.07.2021). Informed consent for study participation and data publication was obtained from each patient or their legal guardian.

We enrolled 82 healthy volunteers as the control group. Informed consent for study participation and data publication was obtained from each volunteer, and the General Data Protection Regulation was adhered to.

### 4.2. Study Cohort

Participants included individuals aged 18 and above diagnosed with sepsis or septic shock according to the Sepsis-3 Consensus criteria [1]. The exclusion criteria were as follows: current cancer with chemotherapy or radiation therapy, ongoing treatment with corticosteroids or immunosuppressive medication, and evidence of autoimmune disorders.

We divided the patients into four subcategories according to diagnostic (sepsis and septic shock) and survival status (survivors and non-survivors).

Eighty-two healthy volunteers were studied alongside the patients as a control group to assess the cutoff values of IL-7. The control group comprised unrelated individuals (24 males, 58 females) with an average age of 32 years and without known infections in the previous 18 months.

### 4.3. Evaluated Parameters

The parameters under examination included age, gender, complete blood count (CBC), T-helper lymphocytes (CD4+), T-cytotoxic lymphocytes (CD8+), natural killer T CD3+ lymphocytes (NKT), programmed cell death-1 (PD-1), programmed cell death-1 ligand (PD-L1), and interleukin-7 (IL-7). We assessed the parameters on the first and fifth days after confirmation of either sepsis or septic shock in the intensive care unit (ICU), aligning with the findings of the IRIS-7 trial [33].

Venous blood samples were collected from each study participant via peripheral puncture using 10 mL syringes. Blood was collected in K2 EDTA tubes for leukocyte subset identification and sent for processing. For PD-1/PD-L1 expression quantification, blood was collected in clot activator tubes, centrifuged for 15 min at 750 × g, aliquoted, and stored at −80 °C in 1.5 mL Eppendorf tubes until all patients were recruited.

### 4.4. CBC and Lymphocyte Subsets

For CBC and immunophenotyping, venous blood samples were collected in K2 EDTA tubes. The identification of leukocyte subsets was performed using the BD FACSCalibur™ flow cytometer analyser (Becton, Dickinson and Company, Franklin Lakes, NJ, USA), and the interpretation of data was performed with BD CellQuest™ Pro (Becton, Dickinson and Company, Franklin Lakes, NJ, USA). The subsets of leukocytes were expressed as % of total peripheral blood mononuclear cells. We gated on lymphocytes using the Side Scatter/CD45 density plot.

The following combination of monoclonal antibodies conjugated to specific fluorochromes from BD^®^ Biosciences reagents (Becton, Dickinson and Company, Franklin Lakes, NJ, USA) was used:

- CD4/CD8/CD3 (BDTritest, Cat. No. 342414) to identify and enumerate the following T-lymphocyte subset populations: CD3+ T lymphocytes, CD3+CD4+ helper/inducer T lymphocytes, and CD3+CD8+ suppressor/cytotoxic T lymphocytes.
- CD3/CD16+CD56/CD45/CD19 (BD Multitest, Cat. No. 342416) to identify and enumerate the following T-, B-, and NK-lymphocyte subset populations: CD3+ T lymphocytes, CD19+ B lymphocytes, and CD3-CD16+CD56+ NK lymphocytes.

Lymphocytes were identified using CD45 and side scatter (SSC) gating strategy as CD45^bright^ with low SSC. The relative proportion of B (CD3-CD19+) and T (CD3+CD19-) cells was then quantified as the plot was split into four quadrants, allowing the identification of the cells’ single positive for each marker. Then, T cells were identified by CD3+ expression, and CD8+ cytotoxic and CD4+ helper T cells were identified by a CD8 versus CD4 dot plot.

### 4.5. PD-1/PD-L1

The cytokine/target proteins PD-1 and PD-L1 levels were assessed using an ELISA protocol. Commercial kits designed for detecting recombinant and natural human programmed cell death-1 and programmed cell death-1 ligand were employed following the manufacturer’s instructions (EIAab Science Inc., Wuhan, China) and processed using automated ELISA equipment (Dynex DSX, Dynex Technology USA, Chantilly, Virginia, VA, USA). Blood samples were collected via venous puncture using clot activator tubes, then centrifuged for 15 min at 750× *g*. The serum was subsequently aliquoted and stored at −80 °C in 1.5 mL Eppendorf tubes until all patients were recruited.

The ELISA plates used for PD-1/PD-L1 quantification were precoated with antibodies with specificity to PD-1 or PD-L1 protein, respectively. Samples and standards with known concentrations were incubated with a biotin-conjugated antibody for 2 h at 37 °C. During this step, the antigen–antibody complex was formed. After the subsequent wash, avidin conjugated with horseradish peroxidase was added to all wells, followed by 1 h incubation at 37 °C. The second washing step, followed by the addition of the 3,3′, 5,5′-tetramethylbenzidine substrate, allowed the formation of immune complexes to be revealed.

The concentration of the target protein, either PD-1 or PD-L1, was proportional to the intensity of the colour developed in each reaction well; the change in the colour intensity was measured at 450 nm. The software generated a 4-parameter logistic curve, allowing the quantification of the target proteins in the neat serum samples. The detection range for PD-1 was 0.156–10 ng/mL, sensitivity was <0.067 ng/mL, and the coefficients of variations were ≤4.2% for intra-assay and ≤8.7% for inter-assay precision. For PD-L1, the detection range was 0.312–20 ng/mL, sensitivity was <0.098 ng/mL, and the coefficients of variations were ≤6% for intra-assay and ≤7.9% for inter-assay precision.

### 4.6. IL-7 Quantification

For IL-7 detection in serum samples, the ELISA sandwich immunoassay from Abclonal (Abclonal Technology. Co., Ltd., Cummings Park, Woburn, MA, USA) followed the manufacturer’s instructions. Briefly, neat serum samples and calibrators with known concentrations were incubated for 2 h in precoated wells with anti-IL-7 antibodies. The captured antibodies have specificity for both natural and recombinant human IL-7.

After a washing step, a second 1 h incubation with a secondary biotin-conjugated antibody was performed. The addition of streptavidin–horseradish peroxidase and subsequent 3,3′, 5,5′-tetramethylbenzidine substrate revealed the antigen–antibody complexes by changing the colours in the wells. The concentration was directly related to the intensity of the colour, which was read spectrophotometrically at 450 nm. The sample concentration was evaluated using the 4PL calibration curve performed by the Dynex DSX instrument.

The measuring range for IL-7 was 7.8–500 pg/mL, and the minimum detectable concentration was 3.9 pg/mL. The precision was <10% for intra-assay and <15% for inter-assay.

### 4.7. Statistical Analysis

The information was inputted into MS Excel (Microsoft^®^ Excel^®^ for Microsoft 365 MSO Version 2406 Build 16.0.17726.20078, Microsoft Corporation, Redmond, WA, USA). Subsequent statistical analyses, encompassing descriptive and inferential processing, were conducted using GraphPad Prism version 8.4.3 (686), released on 10 June 2020 (GraphPad Software, San Diego, CA, USA). Descriptive statistics included the computation of means or medians with corresponding confidence intervals. The Kolmogorov–Smirnov test was performed to verify the normality of the data distribution. The mean was determined for data exhibiting a normal distribution, while for non-Gaussian distributions, the median and the interquartile range were calculated. We used either the Student’s *t*-test or the Wilcoxon test for paired data, depending on whether the distribution was Gaussian or non-Gaussian. For receiver operating characteristic (ROC) analysis, GraphPad Prism version 8.4.3 (686) was used. *p* ≤ 0.05 was considered to indicate a statistically significant difference.

## 5. Conclusions

Monitoring lymphocyte levels shows potential as a biomarker for tracking sepsis progression. In our study, we noted a mild increase in lymphocyte percentages among both septic and surviving patients. However, lymphocyte percentages alone have not yet been validated as a consistent predictor of sepsis outcomes.

The study emphasises the significance of monitoring IL-7 levels in patients with sepsis and septic shock; low levels were linked to a higher risk of mortality, as seen in the non-survivor group. IL-7 plays a dual role in sepsis; it can also be used to guide exogen therapy with IL-7.

PD-1/PD-L1 axis is a biomarker of the immune system; its upregulated expression weakens costimulatory signalling, resulting in reduced T cell response capacity, lymphopenia, higher mortality rates, and greater vulnerability to nosocomial infections. The immunosuppressive state was particularly evident in the non-survivor group of patients, as indicated by the correlations with both helper and cytotoxic lymphocytes.

## Figures and Tables

**Table 1:** Comparison between median and IQR (interquartile range) values of studied groups with normal lymphocyte range.

		Median (IQR)		*p* ^a^ Value	Normal Range	
		Day 1	Day 5		%	×10^3^/μL
Sepsis	%	0.067 (0.0589)	0.085 (0.088)	**0.0032**	0.205–0.45	1.2–3.4
	×10^3^/μL	0.910 (0.405)	1.02 (0.77)	0.9323		
Septic shock	%	0.0502 (0.0565)	0.064 (0.0774)	0.2387		
	×10^3^/μL	0.86 (0.6605)	1.020 (0.8)	0.1089		
Survivors	%	0.0652 (0.058)	0.116 (0.113)	**0.0014**		
	×10^3^/μL	0.8985 (0.942)	1.035 (1.718)	0.6322		
Non-survivors	%	0.057 (0.06)	0.0635 (0.0537)	0.1316		
	×10^3^/μL	0.91 (0.67)	1.015 (0.7605)	0.5014		

Legend: ^a^ Wilcoxon test. Bold type indicates significance. Normal range measured by the laboratory of the County Emergency Clinical Hospital in Târgu Mureș, Romania.

**Table 2:** Comparative IL-7 values (median and IQR (interquartile range)) of the studied categories of patients with the cutoff value.

	Median (IQR) (pg/mL)		IL-7 Cutoff Value (pg/mL)
	Day 1	Day 5	
Sepsis	3.459 (4.204)	2.382 (2.189)	1.94
Septic shock	5.415 (5.35)	3.459 (4.052)	
Survivors	3.921 (4.346)	3.227 (3.037)	
Non-survivors	4.381 (5.834)	2.542 (2.686)	

**Table 3:** Correlations between lymphocyte subsets on day 1 and day 5 for survivor patients.

		Tc Cells (CD8+), %	NKT (CD3+), %	B Cells (CD19+), %
Th cells (CD4+), %	Day 1	r = −0.9368 (−0.9726 to −0.8575) ***p* ^b^ < 0.0001**	r = −0.4108 (−0.7095 to 0.01305) *p* ^b^ = 0.0575	r = 0.3740 (−0.04725 to 0.6823) *p* ^a^ = 0.0718
	Day 5	r = −0.9788 (−0.9922 to −0.9426) ***p* ^b^ < 0.0001**	r = −0.6998 (−0.8833 to −0.3302) ***p* ^b^ = 0.0018**	r = 0.6929 (0.3004 to 0.8847) ***p* ^b^ = 0.0029**
Tc cells (CD8+), %	Day 1		r = 0.5205 (0.1267 to 0.7726) ***p* ^b^ = 0.0130**	r = −0.4517 (−0.7293 to −0.04645) ***p* ^a^ = 0.0267**
	Day 5		r = 0.6379 (0.2269 to 0.8561) ***p* ^b^ = 0.0059**	r = −0.6794 (−0.8791 to −0.2770) ***p* ^b^ = 0.0038**
NKT (CD3+), %	Day 1			r = −0.4864 (−0.7592 to −0.06832) ***p* ^a^ = 0.0217**
	Day 5			r = −0.4824 (−0.7976 to 0.03964) *p* ^b^ = 0.0686

Legend: ^a^ Spearman test, ^b^ Pearson test. Bold type indicates significance. B cells: B CD19+ lymphocytes, CD: cluster of differentiation, NKT CD3+: natural killer T CD3+ lymphocytes, Tc cells: T cytotoxic CD8+ lymphocytes, Th cells: T helper CD4+ lymphocytes.

**Table 4:** Correlations between lymphocyte subsets on day 1 and day 5 for non-survivor patients.

		Tc Cells (CD8+), %	NKT (CD3+), %	B Cells (CD19+), %
Th cells (CD4+), %	Day 1	r = −0.7114 (−0.8155 to −0.5629) ***p* ^b^ < 0.0001**	r = −0.6415 (−0.7722 to −0.4588) ***p* ^a^ < 0.0001**	r = 0.1206 (−0.1451 to 0.3700) *p* ^a^ = 0.3588
	Day 5	r = −0.9461 (−0.9730 to −0.8939) ***p* ^b^ < 0.0001**	r = −0.4962 (−0.7145 to −0.1899) ***p* ^b^ = 0.0028**	r = 0.1412 (−0.2168 to 0.4657) *p* ^a^ = 0.4257
Tc cells (CD8+), %	Day 1		r = 0.5488 (0.3379 to 0.7072) ***p* ^a^ < 0.0001**	r = −0.1183 (−0.3680 to 0.1474) *p* ^a^ = 0.3682
	Day 5		r = 0.4641 (0.1493 to 0.6934) ***p* ^b^ = 0.0057**	r = −0.08319 (−0.4184 to 0.2720) *p* ^a^ = 0.6400
NKT (CD3+), %	Day 1			r = −0.2894 (−0.5153 to −0.02578) ***p* ^a^ = 0.0276**
	Day 5			r = −0.2814 (−0.5728 to 0.07306) *p* ^a^ = 0.1069

Legend: ^a^ Spearman test, ^b^ Pearson test. Bold type indicates significance. B cells: B CD19+ lymphocytes, CD: cluster of differentiation, NKT CD3+: natural killer T CD3+ lymphocytes, Tc cells: T cytotoxic CD8+ lymphocytes, Th cells: T helper CD4+ lymphocytes.

**Table 5:** Correlations between lymphocyte subsets and the studied parameters on day 1 and day 5 for non-survivor patients.

Parameters (on Day 1)	Th Cells (CD4+), %	Tc Cells (CD8+), %	NKT (CD3+), %	B Cells (CD19+), %
PD-1, ng/mL	r = 0.3463 (0.08886 to 0.5603) ***p* ^a^ = 0.0078**	r = −0.3873 (−0.5920 to −0.1357) ***p* ^a^ = 0.0027**	r = −0.3663 (−0.5775 to −0.1091) ***p* ^a^ = 0.0051**	r = 0.1884 (−0.08889 to 0.4386) *p* ^a^ = 0.1683
PD-L1, ng/mL	r = 0.1843 (−0.08264 to 0.4265) *p* ^b^ = 0.1740	r = −0.2790 (−0.5049 to −0.01736) ***p* ^b^ = 0.0373**	r = 0.003428 (−0.2696 to 0.2759) *p* ^a^ = 0.9802	r = −0.1443 (−0.4035 to 0.1364) *p* ^a^ = 0.2979
IL-7, pg/mL	r = 0.1172 (−0.1484 to 0.3671) *p* ^a^ = 0.3723	r = −0.1029 (−0.3545 to 0.1625) *p* ^a^ = 0.4339	r = −0.05624 (−0.3171 to 0.2125) *p* ^a^ = 0.6750	r = 0.1398 (−0.1307 to 0.3908) *p* ^a^ = 0.2954
Parameter (on day 5)	Th cells (CD4+), %	Tc cells (CD8+), %	NKT (CD3+), %	B cells (CD19+), %
PD-1, ng/mL	r = −0.4123 (−0.6749 to −0.05702) ***p* ^a^ = 0.0212**	r = 0.3414 (−0.02566 to 0.6273) *p* ^a^ = 0.0602	r = 0.05467 (−0.3155 to 0.4104) *p* ^a^ = 0.7702	r = −0.1472 (−0.4851 to 0.2289) *p* ^a^ = 0.4294
PD-L1, ng/mL	r = −0.2116 (−0.5536 to 0.1915) *p* ^b^ = 0.2995	r = 0.2440 (−0.1584 to 0.5768) *p* ^b^ = 0.2297	r = −0.1061 (−0.4739 to 0.2934) *p* ^b^ = 0.6061	r = −0.02838 (−0.4212 to 0.3734) *p* ^a^ = 0.8906
IL-7, pg/mL	r = −0.1133 (−0.4757 to 0.2819) *p* ^a^ = 0.5659	r = 0.1375 (−0.2592 to 0.4944) *p* ^a^ = 0.4855	r = 0.1150 (−0.2803 to 0.4770) *p* ^a^ = 0.5600	r = −0.1754 (−0.5233 to 0.2225) *p* ^a^ = 0.3719
		PD-1, ng/mL on day 1	PD-1, ng/mL on day 5	
	IL-7, pg/mL	r = 0.2317 (−0.04387 to 0.4744) *p* ^a^ = 0.0888	r = 0.3774 (0.001264 to 0.6600) ***p* ^a^ = 0.0436**	

Legend: ^a^ Spearman test, ^b^ Pearson test. Bold type indicates significance. B cells: B CD19+ lymphocytes, CD: cluster of differentiation, IL-7: interleukin-7, NKT CD3+: natural killer T CD3+ lymphocytes, PD-1: programmed cell death protein 1, PD-L1: programmed death ligand 1, Tc cells: T cytotoxic CD8+ lymphocytes, Th cells: T helper CD4+ lymphocytes.

## Data Availability

The data generated in the present study may be requested from the corresponding author.

## Supplementary Materials

The supporting information can be downloaded at: https://www.mdpi.com/article/10.3390/ijms252312612/s1.
