# Supplementary material for: The Role of PD-1/PD-L1 and IL-7 in Lymphocyte Dynamics and Sepsis Progression: A Biomarker Study in Critically Ill Patients
Source: Int J Mol Sci. 2024 Nov 24;25(23):12612. doi: 10.3390/ijms252312612 (PMC11641135; doi:10.3390/ijms252312612)
Supplement: Supplementary file 1 [file ijms-25-12612-s001.zip › ijms-3274149-supplementary.pdf]

Supplementary Materials for "The role of serum-soluble PD-1/PD-L1 in reflecting lymphocyte subsets apoptosis in sepsis"

Table S1. Pathology and infectious site for the entire amount of patients.

**Underlying conditions**	**Number of patients**	**%**	**Infectious site**	**Number of patients**	**%**
Cardiovascular disease	73	83.91	Pulmonary	51	58.6
Renal disease	58	69.88	Abdominal	30	34.5
Respiratory disease	55	63.22	Urinary tract	10	11.5
Neurological disease	42	48.28	Cutaneous	7	8.0
Diabetes	27	31.03	Thoracic cavity	1	1.1
Trauma	7	8.05	Soft tissue	1	1.1
Other	85	97.70	Unidentified	1	1.1

Table S2. Descriptive statistics for the entire amount of patients (N = 87).

	Lymphocyte D1 (%)	Lymphocyte D5 (%)	CD4+ D1 (%)	CD4+ D5 (%)	CD8+ D1 (%)	CD8+ D5 (%)	NKT CD3+ D1 (%)	NKT CD3+ D5 (%)
Number of values	87	52	87	52	87	52	83	51
Minimum	0.01800	0.008000	0.2380	0.2800	0.04200	0.04800	0.000	0.000
25% Percentile	0.03800	0.04300	0.5300	0.5575	0.2040	0.2343	0.02500	0.02300
Median	0.06300	0.07450	0.6300	0.6670	0.2930	0.3000	0.06000	0.06000
75% Percentile	0.09500	0.1255	0.7480	0.7433	0.4000	0.3833	0.1110	0.1090
Maximum	0.5580	0.3170	0.9300	0.9440	0.6880	0.5670	0.5380	0.3200
Range	0.5400	0.3090	0.6920	0.6640	0.6460	0.5190	0.5380	0.3200
Mean	0.07919	0.09390	0.6346	0.6540	0.3070	0.3074	0.07831	0.07559
Std. Deviation	0.07340	0.06908	0.1467	0.1345	0.1292	0.1199	0.07814	0.06811
Std. Error of Mean	0.007869	0.009580	0.01573	0.01865	0.01385	0.01662	0.008576	0.009538
Coefficient of variation	92.69%	73.57%	23.12%	20.56%	42.09%	38.99%	99.77%	90.11%
Skewness	4.160	1.447	-0.3265	-0.3562	0.3635	0.3533	3.067	1.658
Kurtosis	22.87	2.165	-0.1313	0.2361	-0.3064	-0.02421	14.69	3.620

	CD19+ D1 (%)	CD19+ D5 (%)	PD-1 D1 (ng/ml)	PD-1 D5 (ng/ml)	PD-L1 D1 (ng/ml)	PD-L1 D5 (ng/ml)	IL-7 D1 (pg/ml)	IL-7 D5 (pg/ml)
Number of values	84	50	82	49	76	43	83	46
Minimum	0.01000	0.001000	0.01200	0.01200	0.3830	0.7700	0.1620	0.008000

25% Percentile	0.08025	0.05775	0.1025	0.09900	3.585	4.135	1.831	1.438
Median	0.1350	0.1030	0.1820	0.1690	6.293	5.644	4.307	2.866
75% Percentile	0.2168	0.1750	0.2618	0.2665	8.532	7.742	6.666	4.047
Maximum	0.7300	0.5200	1.256	1.055	24.58	26.06	37.43	15.33
Range	0.7200	0.5190	1.244	1.043	24.19	25.29	37.27	15.33
Mean	0.1713	0.1442	0.2358	0.2152	6.349	6.278	5.087	3.574
Std. Deviation	0.1377	0.1241	0.2123	0.1932	4.063	4.183	5.055	3.207
Std. Error of Mean	0.01503	0.01755	0.02344	0.02760	0.4661	0.6380	0.5549	0.4728
Coefficient of variation	80.39%	86.09%	90.04%	89.75%	63.99%	66.63%	99.37%	89.72%
Skewness	1.643	1.361	2.440	2.205	1.438	2.529	3.680	1.888
Kurtosis	3.191	1.311	7.378	6.571	4.861	11.12	20.50	3.741

Legend: CD: cluster of differentiation, CD4+: T helper CD4+ lymphocytes, CD8+: T cytotoxic CD8+ lymphocytes, CD19+: B CD19+ lymphocytes, D1: day 1, D5: day 5, IL-7: interleukin-7, NKT CD3+: natural killer T lymphocytes, PD-1: Programmed cell death protein 1, PD-L1: Programmed death ligand 1.

Table S3. Number of patients with IL-7 values above cut-off value.

	**No. of patients with IL-7 values > IL-7 cut-off (1.94 pg/ml)**	
	Day 1	Day 5
Sepsis (D1 = 57/ D5 = 31)	37	20
Septic shock (D1 = 30/ D5 = 15)	24	11
Survivors (D1 = 24/ D5 = 16)	18	13
Non-survivors (D1 = 63/ D5 = 30)	42	18

Legend: D1: day 1; D5: day 5, IL-7: interleukin-7.

Table S4. Descriptive statistics for sepsis patients (N = 57).

	Lymphocyte D1 (%)	Lymphocyte D5 (%)	CD4+ D1 (%)	CD4+ D5 (%)	CD8+ D1 (%)	CD8+ D5 (%)	NKT CD3+ D1 (%)	NKT CD3+ D5 (%)
Number of values	57	35	57	35	57	35	54	34
Minimum	0.01800	0.01900	0.2730	0.2800	0.06400	0.07200	0.000	0.000
25% Percentile	0.04255	0.04700	0.5245	0.5540	0.2100	0.2380	0.02650	0.02975
Median	0.06700	0.08500	0.6040	0.6500	0.3450	0.3000	0.06650	0.06450
75% Percentile	0.1015	0.1350	0.7490	0.7460	0.4285	0.4140	0.1133	0.1160
Maximum	0.5580	0.3170	0.9120	0.9240	0.6880	0.5670	0.5380	0.3200
Range	0.5400	0.2980	0.6390	0.6440	0.6240	0.4950	0.5380	0.3200
Mean	0.08838	0.09849	0.6238	0.6427	0.3302	0.3232	0.08470	0.08315

Std. Deviation	0.08551	0.06668	0.1462	0.1386	0.1350	0.1269	0.08951	0.07336
Std. Error of Mean	0.01133	0.01127	0.01936	0.02343	0.01789	0.02144	0.01218	0.01258
Coefficient of variation	96.75%	67.70%	23.43%	21.57%	40.90%	39.25%	105.7%	88.23%
Skewness	3.772	1.331	-0.05962	-0.4373	0.1662	0.3081	2.959	1.783
Kurtosis	17.44	1.963	-0.4843	0.1959	-0.4361	-0.4606	12.38	3.613

	CD19+ D1 (%)	CD19+ D5 (%)	PD-1 D1 (ng/ml)	PD-1 D5 (ng/ml)	PD-L1 D1 (ng/ml)	PD-L1 D5 (ng/ml)	IL-7 D1 (pg/ml)	IL-7 D5 (pg/ml)
Number of values	55	33	53	32	49	27	54	31
Minimum	0.01000	0.01700	0.01200	0.01300	0.3830	0.7700	0.2580	0.008000
25% Percentile	0.06800	0.05750	0.1080	0.08475	2.945	4.624	1.793	1.353
Median	0.1200	0.08700	0.1600	0.1435	5.523	5.825	3.459	2.382
75% Percentile	0.2000	0.1565	0.2335	0.2055	7.895	8.524	5.997	3.542
Maximum	0.7300	0.4780	0.7460	0.6310	18.65	26.06	13.30	11.19
Range	0.7200	0.4610	0.7340	0.6180	18.26	25.29	13.05	11.18
Mean	0.1605	0.1236	0.2042	0.1881	5.749	6.733	4.092	3.047
Std. Deviation	0.1424	0.1069	0.1488	0.1600	3.626	4.667	2.963	2.591
Std. Error of Mean	0.01921	0.01861	0.02044	0.02829	0.5180	0.8981	0.4032	0.4654
Coefficient of variation	88.76%	86.52%	72.86%	85.10%	63.07%	69.31%	72.42%	85.05%
Skewness	2.058	1.796	2.004	1.538	0.8086	2.775	1.056	1.758
Kurtosis	4.967	3.308	4.313	1.593	1.836	11.27	0.8093	3.392

Legend: CD: cluster of differentiation, CD4+: T helper CD4+ lymphocytes, CD8+: T cytotoxic CD8+ lymphocytes, CD19+: B CD19+ lymphocytes, D1: day 1, D5: day 5, IL-7: interleukin-7, NKT CD3+: natural killer T lymphocytes, PD-1: Programmed cell death protein 1, PD-L1: Programmed death ligand 1.

Table S5. Descriptive statistics for septic shock patients (N = 30).

	Lymphocyte D1 (%)	Lymphocyte D5 (%)	CD4+ D1 (%)	CD4+ D5 (%)	CD8+ D1 (%)	CD8+ D5 (%)	NKT CD3+ D1 (%)	NKT CD3+ D5 (%)
Number of values	30	17	30	17	30	17	29	17
Minimum	0.02000	0.008000	0.2380	0.4400	0.04200	0.04800	0.005000	0.000
25% Percentile	0.03175	0.03100	0.5863	0.5970	0.1975	0.2230	0.02350	0.01250
Median	0.05025	0.06400	0.6795	0.6780	0.2410	0.2600	0.05500	0.02900
75% Percentile	0.08833	0.1084	0.7435	0.7510	0.3235	0.3130	0.1065	0.1000

Maximum	0.1690	0.3070	0.9300	0.9440	0.5040	0.4940	0.2050	0.1770
Range	0.1490	0.2990	0.6920	0.5040	0.4620	0.4460	0.2000	0.1770
Mean	0.06174	0.08446	0.6550	0.6774	0.2629	0.2748	0.06641	0.06047
Std. Deviation	0.03711	0.07500	0.1481	0.1262	0.1059	0.09956	0.04990	0.05508
Std. Error of Mean	0.006775	0.01819	0.02704	0.03062	0.01933	0.02415	0.009265	0.01336
Coefficient of variation	60.11%	88.80%	22.61%	18.64%	40.28%	36.23%	75.13%	91.08%
Skewness	1.236	1.885	-0.8777	-0.03258	0.5444	-0.02652	0.9797	0.6454
Kurtosis	1.121	4.097	1.290	0.3354	0.2705	1.488	0.5618	-0.7061

	CD19+ D1 (%)	CD19+ D5 (%)	PD-1 D1 (ng/ml)	PD-1 D5 (ng/ml)	PD-L1 D1 (ng/ml)	PD-L1 D5 (ng/ml)	IL-7 D1 (pg/ml)	IL-7 D5 (pg/ml)
Number of values	29	17	29	17	27	16	29	15
Minimum	0.01000	0.001000	0.02700	0.01200	0.9890	1.128	0.1620	0.6890
25% Percentile	0.08050	0.05350	0.09600	0.1170	5.127	3.557	3.032	1.687
Median	0.1730	0.1550	0.2160	0.2070	6.967	4.998	5.415	3.459
75% Percentile	0.2690	0.2905	0.3475	0.3330	9.257	7.502	8.382	5.739
Maximum	0.5150	0.5200	1.256	1.055	24.58	12.30	37.43	15.33
Range	0.5050	0.5190	1.229	1.043	23.59	11.18	37.27	14.64
Mean	0.1918	0.1842	0.2934	0.2664	7.437	5.510	6.941	4.665
Std. Deviation	0.1281	0.1475	0.2897	0.2410	4.630	3.202	7.263	4.096
Std. Error of Mean	0.02379	0.03576	0.05379	0.05845	0.8911	0.8005	1.349	1.058
Coefficient of variation	66.81%	80.06%	98.74%	90.46%	62.25%	58.11%	104.6%	87.80%
Skewness	0.8223	0.8056	1.914	2.375	1.909	0.4911	2.973	1.627
Kurtosis	0.06623	-0.05291	3.817	7.134	6.409	-0.2081	11.17	2.233

Legend: CD: cluster of differentiation, CD4+: T helper CD4+ lymphocytes, CD8+: T cytotoxic CD8+ lymphocytes, CD19+: B CD19+ lymphocytes, D1: day 1, D5: day 5, IL-7: interleukin-7, NKT CD3+: natural killer T lymphocytes, PD-1: Programmed cell death protein 1, PD-L1: Programmed death ligand 1.

Table S6. Comparison of the studied parameters between sepsis and septic shock patients on day 1 and day 5 (median value and IQR).

Parameter	Sepsis		p^a value	Septic shock		p^a value
	Day 1	Day 5		Day 1	Day 5	
Th cells (CD4+), %	0.6040 (0.2245)	0.6500 (0.192)	0.7493	0.6795 (0.1572)	0.6780 (0.154)	0.2683

Tc cells (CD8+), %	0.3450 (0.2185)	0.3000 (0.176)	0.8683	0.2410 (0.126)	0.2600 (0.09)	0.5477
NKT (CD3+), %	0.06650 (0.0868)	0.06450 (0.08625)	0.6214	0.05500 (0.083)	0.02900 (0.0875)	0.2582
B cells (CD19+), %	0.1200 (0.132)	0.08700 (0.099)	0.4624	0.1730 (0.1885)	0.1550 (0.237)	0.3964
PD-1, ng/ml	0.1600 (0.1255)	0.1435 (0.12075)	0.3393	0.2160 (0.2785)	0.2070 (0.216)	>0.9999
PD-L1, ng/ml	5.523 (4.95)	5.825 (3.9)	0.8613	6.967 (4.13)	4.998 (3.945)	**0.0353**
IL-7, pg/ml	3.459 (4.204)	2.382 (2.189)	0.1557	5.415 (5.35)	3.459 (4.052)	0.0946

Legend: [a]Wilcoxon test. Bold type indicates significance. B cells: B CD19+ lymphocytes, CD: cluster of differentiation, IL-7: interleukin-7, NKT CD3+: natural killer T CD3+ lymphocytes, PD-1: programmed cell death protein 1, PD-L1: programmed death ligand 1, Tc cells: T cytotoxic CD8+ lymphocytes, Th cells: T helper CD4+ lymphocytes.

Table S7. Correlations for the lot of sepsis patients on day 1 and day 5.

		Tc cells (CD8+), %	NKT (CD3+), %	B cells (CD19+), %	PD-1, ng/ml	PD-L1, ng/ml	IL-7, pg/ml
Th cells (CD4+), %	Day 1	r = -0.8654 (-0.9188 to -0.7809) **p^b <0.0001**	r = -0.5418 (-0.7110 to -0.3132) **p^a <0.0001**	r = 0.1048 (-0.1729 to 0.3670) p^a = 0.4466	r = 0.1386 (-0.1449 to 0.4010) p^a = 0.3224	r = 0.1374 (-0.1496 to 0.4030) p^b = 0.3466	r = 0.03404 (-0.2435 to 0.3064) p^a = 0.8069
	Day 5	r = -0.9776 (-0.9887 to -0.9557) **p^b <0.0001**	r = -0.5652 (-0.7628 to -0.2710) **p^a = 0.0005**	r = 0.3342 (-0.02090 to 0.6144) p^a = 0.0573	r = -0.2788 (-0.5881 to 0.1016) p^a = 0.1357	r = -0.001538 (-0.4068 to 0.4042) p^a = 0.9942	r = -0.1704 (-0.5138 to 0.2200) p^a = 0.3767
Tc cells (CD8+), %	Day 1		r = 0.6355 (0.4367 to 0.7752) **p^a <0.0001**	r = -0.2397 (-0.4810 to 0.03537) p^a = 0.0780	r = -0.1885 (-0.4431 to 0.09433) p^a = 0.1765	r = -0.2057 (-0.4603 to 0.08011) p^b = 0.1562	r = -0.07636 (-0.3444 to 0.2032) p^a = 0.5831
	Day 5		r = 0.5217 (0.2130 to 0.7358) **p^a = 0.0016**	r = -0.2965 (-0.5877 to 0.06267) p^a = 0.0938	r = 0.2487 (-0.1336 to 0.5665) p^a = 0.1852	r = 0.1078 (-0.3113 to 0.4918) p^a = 0.6081	r = 0.1870 (-0.2036 to 0.5263) p^a = 0.3314
NKT (CD3+), %	Day 1			r = -0.3074 (-0.5412 to -0.02935) **p^a = 0.0267**	r = -0.2421 (-0.4917 to 0.04418) p^a = 0.0869	r = -0.06482 (-0.3502 to 0.2316) p^a = 0.6616	r = -0.1290 (-0.3978 to 0.1601) p^a = 0.3668
	Day 5			r = -0.2532 (-0.5605 to 0.1154) p^a = 0.1620	r = -0.2033 (-0.5384 to 0.1873) p^a = 0.2902	r = -0.2775 (-0.6202 to 0.1541) p^a = 0.1892	r = 0.005203 (-0.3786 to 0.3874) p^a = 0.9790
B cells (CD19+), %	Day 1				r = 0.2205 (-0.06702 to 0.4741) p^a = 0.1201	r = -0.02095 (-0.3111 to 0.2728) p^a = 0.8876	r = 0.2358 (-0.04501 to 0.4821) p^a = 0.0892
	Day 5				r = -0.06000 (-0.4267 to 0.3236) p^a = 0.7572	r = -0.1586 (-0.5450 to 0.2833) p^a = 0.4697	r = -0.04825 (-0.4302 to 0.3484) p^a = 0.8111
	Day 1					r = 0.002837	r = 0.1429

PD-1, ng/ml						(-0.2958 to 0.3010) p^a = 0.9851	(-0.1493 to 0.4122) p^a = 0.3221
	Day 5					r = -0.06957 (-0.4700 to 0.3546) p^a = 0.7467	r = 0.2644 (-0.1320 to 0.5879) p^a = 0.1739
PD-L1, ng/ml	Day 1						r = -0.01214 (-0.3062 to 0.2840) p^a = 0.9354
	Day 5						r = 0.2504 (-0.1824 to 0.6020) p^a = 0.2379

Legend: [a]Spearman test, [b]Pearson test. Bold type indicates significance. B cells: B CD19+ lymphocytes, CD: cluster of differentiation, IL-7: interleukin-7, NKT CD3+: natural killer T CD3+ lymphocytes, PD-1: programmed cell death protein 1, PD-L1: programmed death ligand 1, Tc cells: T cytotoxic CD8+ lymphocytes, Th cells: T helper CD4+ lymphocytes.

,

Table S8. Correlations for the lot of septic shock patients on day 1 and day 5.

		Tc cells (CD8+), %	NKT (CD3+), %	B cells (CD19+), %	PD-1, ng/ml	PD-L1, ng/ml	IL-7, pg/ml
Th cells (CD4+), %	Day 1	r = -0.4830 (-0.7183 to -0.1486) **p^b = 0.0069**	r = -0.7446 (-0.8756 to -0.5117) **p^a <0.0001**	r = 0.1272 (-0.2616 to 0.4805) p^a = 0.5109	r = 0.2340 (-0.1561 to 0.5609) p^a = 0.2219	r = 0.06571 (-0.3224 to 0.4349) p^a = 0.7447	r = 0.3342 (-0.04816 to 0.6311) p^a = 0.0764
	Day 5	r = -0.9042 (-0.9653 to -0.7491) **p^b <0.0001**	r = -0.5611 (-0.8255 to -0.09482) **p^a = 0.0208**	r = 0.08795 (-0.4100 to 0.5455) p^b = 0.7371	r = -0.03431 (-0.5180 to 0.4660) p^a = 0.8984	r = -0.4075 (-0.7514 to 0.1105) p^a = 0.1172	r = -0.2143 (-0.6641 to 0.3495) p^a = 0.4421
Tc cells (CD8+), %	Day 1		r = 0.3034 (-0.08225 to 0.6101) p^a = 0.1096	r = 0.2028 (-0.1878 to 0.5381) p^a = 0.2913	r = -0.3345 (-0.6313 to 0.04784) p^a = 0.0761	r = -0.04149 (-0.4150 to 0.3439) p^a = 0.8372	r = -0.1846 (-0.5244 to 0.2061) p^a = 0.3379
	Day 5		r = 0.2910 (-0.2352 to 0.6852) p^a = 0.2553	r = -0.1008 (-0.5546 to 0.3992) p^b = 0.7002	r = -0.02696 (-0.5126 to 0.4718) p^a = 0.9209	r = 0.4329 (-0.08000 to 0.7645) p^b = 0.0940	r = -0.03214 (-0.5474 to 0.5008) p^a = 0.9132
NKT (CD3+), %	Day 1			r = -0.3174 (-0.6245 to 0.07470) p^a = 0.0998	r = -0.1497 (-0.5038 to 0.2475) p^a = 0.4471	r = 0.2596 (-0.1539 to 0.5957) p^a = 0.2003	r = -0.1346 (-0.4922 to 0.2620) p^a = 0.4948
	Day 5			r = -0.3131 (-0.6979 to 0.2121) p^a = 0.2197	r = 0.01596 (-0.4803 to 0.5045) p^a = 0.9528	r = -0.02653 (-0.5272 to 0.4878) p^a = 0.9234	r = 0.1594 (-0.3984 to 0.6311) p^a = 0.5678
B cells (CD19+), %	Day 1				r = -0.1896 (-0.5339 to 0.2085) p^a = 0.3338	r = -0.3269 (-0.6412 to 0.08117) p^a = 0.1030	r = -0.1188 (-0.4800 to 0.2768) p^a = 0.5471
	Day 5				r = -0.3848 (-0.7375 to 0.1328) p^a = 0.1281	r = -0.05517 (-0.5362 to 0.4529) p^a = 0.8392	r = -0.4857 (-0.8051 to 0.05204) p^a = 0.0688

PD-1, ng/ml	Day 1	r = 0.4566 (0.07218 to 0.7230) **p^a = 0.0190**	r = 0.2940 (-0.1003 to 0.6085) p^a = 0.1288
	Day 5	r = 0.3088 (-0.2359 to 0.7059) p^a = 0.2440	r = 0.5750 (0.07231 to 0.8447) **p^a = 0.0274**
PD-L1, ng/ml	Day 1		r = 0.02154 (-0.3793 to 0.4156) p^a = 0.9168
	Day 5		r = 0.2393 (-0.3261 to 0.6786) p^a = 0.3892

Legend: [a]Spearman test, [b]Pearson test. Bold type indicates significance. B cells: B CD19+ lymphocytes, CD: cluster of differentiation, IL-7: interleukin-7, NKT CD3+: natural killer T CD3+ lymphocytes, PD-1: programmed cell death protein 1, PD-L1: programmed death ligand 1, Tc cells: T cytotoxic CD8+ lymphocytes, Th cells: T helper CD4+ lymphocytes.

Table S9. Descriptive statistics for survivor patients (N = 24).

	Lymphocyte D1 (%)	Lymphocyte D5 (%)	CD4+ D1 (%)	CD4+ D5 (%)	CD8+ D1 (%)	CD8+ D5 (%)	NKT CD3+ D1 (%)	NKT CD3+ D5 (%)
Number of values	24	16	24	18	24	18	22	17
Minimum	0.02900	0.02500	0.4160	0.4340	0.1441	0.1450	0.000	0.006000
25% Percentile	0.04123	0.06850	0.5618	0.5285	0.2065	0.2265	0.03050	0.02150
Median	0.06520	0.1160	0.6635	0.6535	0.2800	0.3060	0.05500	0.08800
75% Percentile	0.09925	0.1815	0.7208	0.7430	0.3908	0.4230	0.09900	0.1230
Maximum	0.1690	0.3070	0.8300	0.8360	0.4710	0.5610	0.1760	0.2940
Range	0.1400	0.2820	0.4140	0.4020	0.3269	0.4160	0.1760	0.2880
Mean	0.07612	0.1266	0.6509	0.6365	0.3005	0.3263	0.06686	0.09041
Std. Deviation	0.04119	0.07542	0.1105	0.1312	0.1006	0.1276	0.04736	0.07270
Std. Error of Mean	0.008409	0.01886	0.02256	0.03092	0.02053	0.03008	0.01010	0.01763
Coefficient of variation	54.12%	59.60%	16.98%	20.61%	33.47%	39.11%	70.83%	80.41%
Skewness	0.9149	0.8203	-0.2240	-0.2087	0.1214	0.4695	0.6784	1.288
Kurtosis	-0.1395	0.6945	-0.5960	-0.9308	-1.424	-0.8223	-0.2097	2.686
	CD19+ D1 (%)	CD19+ D5 (%)	PD-1 D1 (ng/ml)	PD-1 D5 (ng/ml)	PD-L1 D1 (ng/ml)	PD-L1 D5 (ng/ml)	IL-7 D1 (pg/ml)	IL-7 D5 (pg/ml)
Number of values	24	16	24	16	20	15	23	16
Minimum	0.01000	0.02500	0.04400	0.01200	0.9210	0.7700	1.293	0.6380

25% Percentile	0.07775	0.05950	0.09325	0.07750	3.585	4.156	2.199	2.175
Median	0.1170	0.09350	0.1750	0.1880	5.170	5.249	3.921	3.227
75% Percentile	0.1620	0.1573	0.2373	0.3680	7.297	6.808	6.545	5.212
Maximum	0.5680	0.2810	0.9640	0.5510	18.65	26.06	37.43	10.47
Range	0.5580	0.2560	0.9200	0.5390	17.73	25.29	36.14	9.831
Mean	0.1441	0.1123	0.2328	0.2315	5.753	6.505	5.790	4.088
Std. Deviation	0.1177	0.07075	0.2175	0.1787	3.845	5.672	7.320	3.205
Std. Error of Mean	0.02403	0.01769	0.04440	0.04467	0.8597	1.464	1.526	0.8013
Coefficient of variation	81.69%	63.03%	93.42%	77.19%	66.84%	87.20%	126.4%	78.41%
Skewness	2.334	1.015	2.169	0.6842	2.045	3.259	3.973	1.246
Kurtosis	6.946	0.6629	5.069	-0.7186	6.246	11.90	17.44	0.3023

Legend: CD: cluster of differentiation, CD4+: T helper CD4+ lymphocytes, CD8+: T cytotoxic CD8+ lymphocytes, CD19+: B CD19+ lymphocytes, D1: day 1, D5: day 5, IL-7: interleukin-7, NKT CD3+: natural killer T lymphocytes, PD-1: Programmed cell death protein 1, PD-L1: Programmed death ligand 1.

Table S10. Descriptive statistics for non-survivor patients (N = 63).

	Lymphocyte D1 (%)	Lymphocyte D5 (%)	CD4+ D1 (%)	CD4+ D5 (%)	CD8+ D1 (%)	CD8+ D5 (%)	NKT CD3+ D1 (%)	NKT CD3+ D5 (%)
Number of values	63	36	63	34	63	34	61	34
Minimum	0.01800	0.008000	0.2380	0.2800	0.04200	0.04800	0.000	0.000
25% Percentile	0.03500	0.03825	0.5230	0.5625	0.2040	0.2368	0.02450	0.02225
Median	0.05700	0.06350	0.6220	0.6790	0.2930	0.3000	0.06000	0.04900
75% Percentile	0.09500	0.09200	0.7500	0.7468	0.4280	0.3648	0.1155	0.09650
Maximum	0.5580	0.3170	0.9300	0.9440	0.6880	0.5670	0.5380	0.3200
Range	0.5400	0.3090	0.6920	0.6640	0.6460	0.5190	0.5380	0.3200
Mean	0.08036	0.07939	0.6284	0.6633	0.3094	0.2974	0.08244	0.06818
Std. Deviation	0.08270	0.06174	0.1587	0.1372	0.1392	0.1162	0.08656	0.06555
Std. Error of Mean	0.01042	0.01029	0.01999	0.02353	0.01754	0.01993	0.01108	0.01124
Coefficient of variation	102.9%	77.77%	25.26%	20.68%	44.99%	39.09%	105.0%	96.15%
Skewness	3.998	2.028	-0.2709	-0.4637	0.3637	0.2598	2.969	2.004
Kurtosis	19.52	5.296	-0.3186	0.9391	-0.3905	0.5428	12.74	5.566
	CD19+ D1 (%)	CD19+ D5 (%)	PD-1 D1 (ng/ml)	PD-1 D5 (ng/ml)	PD-L1 D1 (ng/ml)	PD-L1 D5 (ng/ml)	IL-7 D1 (pg/ml)	IL-7 D5 (pg/ml)

Number of values	60	34	58	33	56	28	60	30
Minimum	0.01000	0.001000	0.01200	0.01300	0.3830	1.128	0.1620	0.008000
25% Percentile	0.08100	0.04325	0.1030	0.09900	3.338	3.720	1.426	1.361
Median	0.1510	0.1090	0.1855	0.1570	6.964	5.814	4.381	2.542
75% Percentile	0.2465	0.2678	0.2680	0.2270	8.854	8.948	7.260	4.047
Maximum	0.7300	0.5200	1.256	1.055	24.58	12.30	20.90	15.33
Range	0.7200	0.5190	1.244	1.042	24.19	11.18	20.74	15.33
Mean	0.1822	0.1592	0.2370	0.2074	6.562	6.157	4.818	3.300
Std. Deviation	0.1444	0.1410	0.2120	0.2020	4.151	3.240	3.908	3.228
Std. Error of Mean	0.01864	0.02418	0.02783	0.03517	0.5547	0.6124	0.5045	0.5893
Coefficient of variation	79.28%	88.55%	89.45%	97.43%	63.25%	52.63%	81.11%	97.81%
Skewness	1.476	1.100	2.621	2.776	1.307	-0.003926	1.464	2.362
Kurtosis	2.670	0.2709	9.037	9.433	5.075	-0.9375	3.619	6.652

Legend: CD: cluster of differentiation, CD4+: T helper CD4+ lymphocytes, CD8+: T cytotoxic CD8+ lymphocytes, CD19+: B CD19+ lymphocytes, D1: day 1, D5: day 5, IL-7: interleukin-7, NKT CD3+: natural killer T lymphocytes, PD-1: Programmed cell death protein 1, PD-L1: Programmed death ligand 1.

Table S11. Comparison of the studied parameters between survivors and non-survivor patients on day 1 and day 5 (median value and IQR).

Parameter	Survivors		p^a value	Non-survivors		p^a value
	Day 1	Day 5		Day 1	Day 5	
Th cells (CD4+), %	0.6635 (0.159)	0.6535 (0.2145)	0.4233	0.6220 (0.227)	0.6790 (0.1843)	0.9764
Tc cells (CD8+), %	0.2800 (0.1843)	0.3060 (0.1965)	0.2601	0.2930 (0.224)	0.3000 (0.128)	0.9093
NKT (CD3+), %	0.05500 (0.0595)	0.08800 (0.1015)	**0.0076**	0.06000 (0.091)	0.04900 (0.07425)	0.4346
B cells (CD19+), %	0.1170 (0.08425)	0.09350 (0.0978)	0.4884	0.1510 (0.1655)	0.1090 (0.2245)	0.4238
PD-1, ng/ml	0.1750 (0.14405)	0.1880 (0.2905)	0.8603	0.1855 (0.165)	0.1570 (0.128)	0.2929
PD-L1, ng/ml	5.170 (3.712)	5.249 (2.652)	0.5830	6.964 (5.516)	5.814 (5.228)	0.3242
IL-7, pg/ml	3.921 (4.346)	3.227 (3.037)	0.9780	4.381 (5.834)	2.542 (2.686)	**0.0069**

Legend: [a]Wilcoxon test. Bold type indicates significance. B cells: B CD19+ lymphocytes, CD: cluster of differentiation, IL-7: interleukin-7, NKT CD3+: natural killer T CD3+ lymphocytes, PD-1: programmed cell death protein 1, PD-L1: programmed death ligand 1, Tc cells: T cytotoxic CD8+ lymphocytes, Th cells: T helper CD4+ lymphocytes.

Table S12. Correlations for the lot of survivor patients on day 1 and day 5.

		Tc cells (CD8+), %	NKT, %	B cells (CD19+), %	PD-1, ng/ml	PD-L1, ng/ml	IL-7, pg/ml
Th cells (CD4+), %	Day 1	r = -0.9368 (-0.9726 to -0.8575) **p^b <0.0001**	r = -0.4108 (-0.7095 to 0.01305) p^b = 0.0575	r = 0.3740 (-0.04725 to 0.6823) p^a = 0.0718	r = -0.2344 (-0.5910 to 0.1988) p^a = 0.2703	r = 0.1030 (-0.3679 to 0.5319) p^a = 0.6655	r = 0.2753 (-0.1670 to 0.6254) p^a = 0.2035
	Day 5	r = -0.9788 (-0.9922 to -0.9426) **p^b <0.0001**	r = -0.6998 (-0.8833 to -0.3302) **p^b = 0.0018**	r = 0.6929 (0.3004 to 0.8847) **p^b = 0.0029**	r = 0.05296 (-0.4547 to 0.5346) p^b = 0.8456	r = -0.06786 (-0.5720 to 0.4735) p^a = 0.8124	r = 0.02941 (-0.4856 to 0.5292) p^a = 0.9171
Tc cells (CD8+), %	Day 1		r = 0.5205 (0.1267 to 0.7726) **p^b = 0.0130**	r = -0.4517 (-0.7293 to -0.04645) **p^a = 0.0267**	r = 0.1723 (-0.2602 to 0.5472) p^a = 0.4207	r = -0.07446 (-0.5110 to 0.3926) p^a = 0.7550	r = -0.2330 (-0.5971 to 0.2107) p^a = 0.2847
	Day 5		r = 0.6379 (0.2269 to 0.8561) **p^b = 0.0059**	r = -0.6794 (-0.8791 to -0.2770) **p^b = 0.0038**	r = -0.01212 (-0.5048 to 0.4865) p^b = 0.9645	r = 0.1893 (-0.3722 to 0.6493) p^a = 0.4983	r = -0.1176 (-0.5901 to 0.4149) p^a = 0.6645
NKT, %	Day 1			r = -0.4864 (-0.7592 to -0.06832) **p^a = 0.0217**	r = 0.1725 (-0.2809 to 0.5630) p^a = 0.4427	r = -0.07476 (-0.5222 to 0.4050) p^a = 0.7610	r = -0.3839 (-0.7065 to 0.07092) p^a = 0.0858
	Day 5			r = -0.4824 (-0.7976 to 0.03964) p^b = 0.0686	r = -0.4909 (-0.8016 to 0.02859) p^b = 0.0632	r = -0.2992 (-0.7245 to 0.2911) p^a = 0.2965	r = -0.2181 (-0.6663 to 0.3460) p^a = 0.4318
B cells (CD19+), %	Day 1				r = -0.2339 (-0.5907 to 0.1993) p^a = 0.2713	r = -0.07068 (-0.5081 to 0.3958) p^a = 0.7672	r = 0.07660 (-0.3579 to 0.4838) p^a = 0.7283
	Day 5				r = -0.1500 (-0.6150 to 0.3924) p^b = 0.5937	r = -0.1978 (-0.6850 to 0.4117) p^a = 0.5171	r = 0.09011 (-0.4762 to 0.6036) p^a = 0.7616
PD-1, ng/ml	Day 1					r = -0.1835 (-0.5882 to 0.2948) p^a = 0.4388	r = 0.1977 (-0.2458 to 0.5727) p^a = 0.3659
	Day 5					r = 0.05934 (-0.4998 to 0.5836) p^a = 0.8438	r = 0.2484 (-0.3406 to 0.6973) p^a = 0.3911
PD-L1, ng/ml	Day 1						r = 0.1596 (-0.3306 to 0.5820) p^a = 0.5138
	Day 5						r = 0.06786 (-0.4735 to 0.5720) p^a = 0.8124

Legend: [a]Spearman test, [b]Pearson test. Bold type indicates significance. B cells: B CD19+ lymphocytes, CD: cluster of differentiation, IL-7: interleukin-7, NKT CD3+: natural killer T CD3+ lymphocytes, PD-1: programmed cell death protein 1, PD-L1: programmed death ligand 1, Tc cells: T cytotoxic CD8+ lymphocytes, Th cells: T helper CD4+ lymphocytes.

Table S13. Correlations for the lot of non-survivor patients on day 1 and day 5.

		Tc cells (CD8+), %	NKT, %	B cells (CD19+), %	PD-1, ng/ml	PD-L1, ng/ml	IL-7, pg/ml
Th cells (CD4+), %	Day 1	r = -0.7114 (-0.8155 to -0.5629) ***p*^b^ <0.0001**	r = -0.6415 (-0.7722 to -0.4588) ***p*^a^ <0.0001**	r = 0.1206 (-0.1451 to 0.3700) *p*^a^ = 0.3588	r = 0.3463 (0.08886 to 0.5603) ***p*^a^ = 0.0078**	r = 0.1843 (-0.08264 to 0.4265) *p*^b^ = 0.1740	r = 0.1172 (-0.1484 to 0.3671) *p*^a^ = 0.3723
	Day 5	r = -0.9461 (-0.9730 to -0.8939) ***p*^b^ <0.0001**	r = -0.4962 (-0.7145 to -0.1899) ***p*^b^ = 0.0028**	r = 0.1412 (-0.2168 to 0.4657) *p*^a^ = 0.4257	r = -0.4123 (-0.6749 to -0.05702) ***p*^a^ = 0.0212**	r = -0.2116 (-0.5536 to 0.1915) *p*^b^ = 0.2995	r = -0.1133 (-0.4757 to 0.2819) *p*^a^ = 0.5659
Tc cells (CD8+), %	Day 1		r = 0.5488 (0.3379 to 0.7072) ***p*^a^ <0.0001**	r = -0.1183 (-0.3680 to 0.1474) *p*^a^ = 0.3682	r = -0.3873 (-0.5920 to -0.1357) ***p*^a^ = 0.0027**	r = -0.2790 (-0.5049 to -0.01736) ***p*^b^ = 0.0373**	r = -0.1029 (-0.3545 to 0.1625) *p*^a^ = 0.4339
	Day 5		r = 0.4641 (0.1493 to 0.6934) ***p*^b^ = 0.0057**	r = -0.08319 (-0.4184 to 0.2720) *p*^a^ = 0.6400	r = 0.3414 (-0.02566 to 0.6273) *p*^a^ = 0.0602	r = 0.2440 (-0.1584 to 0.5768) *p*^b^ = 0.2297	r = 0.1375 (-0.2592 to 0.4944) *p*^a^ = 0.4855
NKT, %	Day 1			r = -0.2894 (-0.5153 to -0.02578) ***p*^a^ = 0.0276**	r = -0.3663 (-0.5775 to -0.1091) ***p*^a^ = 0.0051**	r = 0.003428 (-0.2696 to 0.2759) *p*^a^ = 0.9802	r = -0.05624 (-0.3171 to 0.2125) *p*^a^ = 0.6750
	Day 5			r = -0.2814 (-0.5728 to 0.07306) *p*^a^ = 0.1069	r = 0.05467 (-0.3155 to 0.4104) *p*^a^ = 0.7702	r = -0.1061 (-0.4739 to 0.2934) *p*^b^ = 0.6061	r = 0.1150 (-0.2803 to 0.4770) *p*^a^ = 0.5600
B cells (CD19+), %	Day 1				r = 0.1884 (-0.08889 to 0.4386) *p*^a^ = 0.1683	r = -0.1443 (-0.4035 to 0.1364) *p*^a^ = 0.2979	r = 0.1398 (-0.1307 to 0.3908) *p*^a^ = 0.2954
	Day 5				r = -0.1472 (-0.4851 to 0.2289) *p*^a^ = 0.4294	r = -0.02838 (-0.4212 to 0.3734) *p*^a^ = 0.8906	r = -0.1754 (-0.5233 to 0.2225) *p*^a^ = 0.3719
PD-1, ng/ml	Day 1					r = 0.2528 (-0.02984 to 0.4980) *p*^a^ = 0.0706	r = 0.2317 (-0.04387 to 0.4744) *p*^a^ = 0.0888
	Day 5					r = 0.03316 (-0.3693 to 0.4251) *p*^a^ = 0.8722	r = 0.3774 (0.001264 to 0.6600) ***p*^a^ = 0.0436**
PD-L1, ng/ml	Day 1						r = 0.008348 (-0.2675 to 0.2830) *p*^a^ = 0.9522
	Day 5						r = 0.2304 (-0.2028 to 0.5883) *p*^a^ = 0.2787

Legend: [a]Spearman test, [b]Pearson test. Bold type indicates significance. B cells: B CD19+ lymphocytes, CD: cluster of differentiation, IL-7: interleukin-7, NKT CD3+: natural killer T CD3+ lymphocytes, PD-1: programmed cell death protein 1, PD-L1: programmed death ligand 1, Tc cells: T cytotoxic CD8+ lymphocytes, Th cells: T helper CD4+ lymphocytes.
