# Supplementary material for: The Role of PD-1/PD-L1 and IL-7 in Lymphocyte Dynamics and Sepsis Progression: A Biomarker Study in Critically Ill Patients
Source: Int J Mol Sci. 2024 Nov 24;25(23):12612. doi: 10.3390/ijms252312612 (PMC11641135; doi:10.3390/ijms252312612)
Supplement: Supplementary file 1 [file ijms-25-12612-s001.zip › Supplementary Materials/Supplementary Materials.docx]

Supplementary Materials for “The role of serum-soluble PD-1/PD-L1 in reflecting lymphocyte subsets apoptosis in sepsis”

**Table S1**. Pathology and infectious site for the entire amount of patients.

| **Underlying conditions** | **Number of patients** | **%** | **Infectious site** | **Number of patients** | **%** |
| --- | --- | --- | --- | --- | --- |
| Cardiovascular disease | 73 | 83.91 | Pulmonary | 51 | 58.6 |
| Renal disease | 58 | 69.88 | Abdominal | 30 | 34.5 |
| Respiratory disease | 55 | 63.22 | Urinary tract | 10 | 11.5 |
| Neurological disease | 42 | 48.28 | Cutaneous | 7 | 8.0 |
| Diabetes | 27 | 31.03 | Thoracic cavity | 1 | 1.1 |
| Trauma | 7 | 8.05 | Soft tissue | 1 | 1.1 |
| Other | 85 | 97.70 | Unidentified | 1 | 1.1 |

**Table S2**. Descriptive statistics for the entire amount of patients (N = 87).

|  | Lymphocyte D1 (%) | Lymphocyte D5 (%) | CD4+  D1 (%) | CD4+  D5 (%) | CD8+  D1 (%) | CD8+  D5 (%) | NKT CD3+  D1 (%) | NKT CD3+  D5 (%) |
| --- | --- | --- | --- | --- | --- | --- | --- | --- |
| Number of values | 87 | 52 | 87 | 52 | 87 | 52 | 83 | 51 |
|  |  |  |  |  |  |  |  |  |
| Minimum | 0.01800 | 0.008000 | 0.2380 | 0.2800 | 0.04200 | 0.04800 | 0.000 | 0.000 |
| 25% Percentile | 0.03800 | 0.04300 | 0.5300 | 0.5575 | 0.2040 | 0.2343 | 0.02500 | 0.02300 |
| Median | 0.06300 | 0.07450 | 0.6300 | 0.6670 | 0.2930 | 0.3000 | 0.06000 | 0.06000 |
| 75% Percentile | 0.09500 | 0.1255 | 0.7480 | 0.7433 | 0.4000 | 0.3833 | 0.1110 | 0.1090 |
| Maximum | 0.5580 | 0.3170 | 0.9300 | 0.9440 | 0.6880 | 0.5670 | 0.5380 | 0.3200 |
| Range | 0.5400 | 0.3090 | 0.6920 | 0.6640 | 0.6460 | 0.5190 | 0.5380 | 0.3200 |
|  |  |  |  |  |  |  |  |  |
| Mean | 0.07919 | 0.09390 | 0.6346 | 0.6540 | 0.3070 | 0.3074 | 0.07831 | 0.07559 |
| Std. Deviation | 0.07340 | 0.06908 | 0.1467 | 0.1345 | 0.1292 | 0.1199 | 0.07814 | 0.06811 |
| Std. Error of Mean | 0.007869 | 0.009580 | 0.01573 | 0.01865 | 0.01385 | 0.01662 | 0.008576 | 0.009538 |
|  |  |  |  |  |  |  |  |  |
| Coefficient of variation | 92.69% | 73.57% | 23.12% | 20.56% | 42.09% | 38.99% | 99.77% | 90.11% |
|  |  |  |  |  |  |  |  |  |
| Skewness | 4.160 | 1.447 | -0.3265 | -0.3562 | 0.3635 | 0.3533 | 3.067 | 1.658 |
| Kurtosis | 22.87 | 2.165 | -0.1313 | 0.2361 | -0.3064 | -0.02421 | 14.69 | 3.620 |
|  |  |  |  |  |  |  |  |  |
|  | CD19+  D1 (%) | CD19+  D5 (%) | PD-1  D1 (ng/ml) | PD-1  D5 (ng/ml) | PD-L1  D1 (ng/ml) | PD-L1  D5 (ng/ml) | IL-7  D1 (pg/ml) | IL-7  D5 (pg/ml) |
| Number of values | 84 | 50 | 82 | 49 | 76 | 43 | 83 | 46 |
|  |  |  |  |  |  |  |  |  |
| Minimum | 0.01000 | 0.001000 | 0.01200 | 0.01200 | 0.3830 | 0.7700 | 0.1620 | 0.008000 |
| 25% Percentile | 0.08025 | 0.05775 | 0.1025 | 0.09900 | 3.585 | 4.135 | 1.831 | 1.438 |
| Median | 0.1350 | 0.1030 | 0.1820 | 0.1690 | 6.293 | 5.644 | 4.307 | 2.866 |
| 75% Percentile | 0.2168 | 0.1750 | 0.2618 | 0.2665 | 8.532 | 7.742 | 6.666 | 4.047 |
| Maximum | 0.7300 | 0.5200 | 1.256 | 1.055 | 24.58 | 26.06 | 37.43 | 15.33 |
| Range | 0.7200 | 0.5190 | 1.244 | 1.043 | 24.19 | 25.29 | 37.27 | 15.33 |
|  |  |  |  |  |  |  |  |  |
| Mean | 0.1713 | 0.1442 | 0.2358 | 0.2152 | 6.349 | 6.278 | 5.087 | 3.574 |
| Std. Deviation | 0.1377 | 0.1241 | 0.2123 | 0.1932 | 4.063 | 4.183 | 5.055 | 3.207 |
| Std. Error of Mean | 0.01503 | 0.01755 | 0.02344 | 0.02760 | 0.4661 | 0.6380 | 0.5549 | 0.4728 |
|  |  |  |  |  |  |  |  |  |
| Coefficient of variation | 80.39% | 86.09% | 90.04% | 89.75% | 63.99% | 66.63% | 99.37% | 89.72% |
|  |  |  |  |  |  |  |  |  |
| Skewness | 1.643 | 1.361 | 2.440 | 2.205 | 1.438 | 2.529 | 3.680 | 1.888 |
| Kurtosis | 3.191 | 1.311 | 7.378 | 6.571 | 4.861 | 11.12 | 20.50 | 3.741 |

Legend: CD: cluster of differentiation, CD4+: T helper CD4+ lymphocytes, CD8+: T cytotoxic CD8+ lymphocytes, CD19+: B CD19+ lymphocytes, D1: day 1, D5: day 5, IL-7: interleukin-7, NKT CD3+: natural killer T lymphocytes, PD-1: Programmed cell death protein 1, PD-L1: Programmed death ligand 1.

**Table S3.** Number of patients with IL-7 values above cut-off value.

|  | **No. of patients with IL-7 values > IL-7 cut-off (1.94 pg/ml)** | |
| --- | --- | --- |
|  | Day 1 | Day 5 |
| Sepsis (D1 = 57/ D5 = 31) | 37 | 20 |
| Septic shock (D1 = 30/ D5 = 15) | 24 | 11 |
| Survivors (D1 = 24/ D5 = 16) | 18 | 13 |
| Non-survivors (D1 = 63/ D5 = 30) | 42 | 18 |

Legend: D1: day 1; D5: day 5, IL-7: interleukin-7.

**Table S4**. Descriptive statistics for sepsis patients (N = 57).

|  | Lymphocyte D1 (%) | Lymphocyte D5 (%) | CD4+  D1 (%) | CD4+  D5 (%) | CD8+  D1 (%) | CD8+  D5 (%) | NKT CD3+  D1 (%) | NKT CD3+  D5 (%) |
| --- | --- | --- | --- | --- | --- | --- | --- | --- |
| Number of values | 57 | 35 | 57 | 35 | 57 | 35 | 54 | 34 |
|  |  |  |  |  |  |  |  |  |
| Minimum | 0.01800 | 0.01900 | 0.2730 | 0.2800 | 0.06400 | 0.07200 | 0.000 | 0.000 |
| 25% Percentile | 0.04255 | 0.04700 | 0.5245 | 0.5540 | 0.2100 | 0.2380 | 0.02650 | 0.02975 |
| Median | 0.06700 | 0.08500 | 0.6040 | 0.6500 | 0.3450 | 0.3000 | 0.06650 | 0.06450 |
| 75% Percentile | 0.1015 | 0.1350 | 0.7490 | 0.7460 | 0.4285 | 0.4140 | 0.1133 | 0.1160 |
| Maximum | 0.5580 | 0.3170 | 0.9120 | 0.9240 | 0.6880 | 0.5670 | 0.5380 | 0.3200 |
| Range | 0.5400 | 0.2980 | 0.6390 | 0.6440 | 0.6240 | 0.4950 | 0.5380 | 0.3200 |
|  |  |  |  |  |  |  |  |  |
| Mean | 0.08838 | 0.09849 | 0.6238 | 0.6427 | 0.3302 | 0.3232 | 0.08470 | 0.08315 |
| Std. Deviation | 0.08551 | 0.06668 | 0.1462 | 0.1386 | 0.1350 | 0.1269 | 0.08951 | 0.07336 |
| Std. Error of Mean | 0.01133 | 0.01127 | 0.01936 | 0.02343 | 0.01789 | 0.02144 | 0.01218 | 0.01258 |
|  |  |  |  |  |  |  |  |  |
| Coefficient of variation | 96.75% | 67.70% | 23.43% | 21.57% | 40.90% | 39.25% | 105.7% | 88.23% |
|  |  |  |  |  |  |  |  |  |
| Skewness | 3.772 | 1.331 | -0.05962 | -0.4373 | 0.1662 | 0.3081 | 2.959 | 1.783 |
| Kurtosis | 17.44 | 1.963 | -0.4843 | 0.1959 | -0.4361 | -0.4606 | 12.38 | 3.613 |
|  |  |  |  |  |  |  |  |  |
|  | CD19+  D1 (%) | CD19+  D5 (%) | PD-1  D1 (ng/ml) | PD-1  D5 (ng/ml) | PD-L1  D1 (ng/ml) | PD-L1  D5 (ng/ml) | IL-7  D1 (pg/ml) | IL-7  D5 (pg/ml) |
| Number of values | 55 | 33 | 53 | 32 | 49 | 27 | 54 | 31 |
|  |  |  |  |  |  |  |  |  |
| Minimum | 0.01000 | 0.01700 | 0.01200 | 0.01300 | 0.3830 | 0.7700 | 0.2580 | 0.008000 |
| 25% Percentile | 0.06800 | 0.05750 | 0.1080 | 0.08475 | 2.945 | 4.624 | 1.793 | 1.353 |
| Median | 0.1200 | 0.08700 | 0.1600 | 0.1435 | 5.523 | 5.825 | 3.459 | 2.382 |
| 75% Percentile | 0.2000 | 0.1565 | 0.2335 | 0.2055 | 7.895 | 8.524 | 5.997 | 3.542 |
| Maximum | 0.7300 | 0.4780 | 0.7460 | 0.6310 | 18.65 | 26.06 | 13.30 | 11.19 |
| Range | 0.7200 | 0.4610 | 0.7340 | 0.6180 | 18.26 | 25.29 | 13.05 | 11.18 |
|  |  |  |  |  |  |  |  |  |
| Mean | 0.1605 | 0.1236 | 0.2042 | 0.1881 | 5.749 | 6.733 | 4.092 | 3.047 |
| Std. Deviation | 0.1424 | 0.1069 | 0.1488 | 0.1600 | 3.626 | 4.667 | 2.963 | 2.591 |
| Std. Error of Mean | 0.01921 | 0.01861 | 0.02044 | 0.02829 | 0.5180 | 0.8981 | 0.4032 | 0.4654 |
|  |  |  |  |  |  |  |  |  |
| Coefficient of variation | 88.76% | 86.52% | 72.86% | 85.10% | 63.07% | 69.31% | 72.42% | 85.05% |
|  |  |  |  |  |  |  |  |  |
| Skewness | 2.058 | 1.796 | 2.004 | 1.538 | 0.8086 | 2.775 | 1.056 | 1.758 |
| Kurtosis | 4.967 | 3.308 | 4.313 | 1.593 | 1.836 | 11.27 | 0.8093 | 3.392 |

Legend: CD: cluster of differentiation, CD4+: T helper CD4+ lymphocytes, CD8+: T cytotoxic CD8+ lymphocytes, CD19+: B CD19+ lymphocytes, D1: day 1, D5: day 5, IL-7: interleukin-7, NKT CD3+: natural killer T lymphocytes, PD-1: Programmed cell death protein 1, PD-L1: Programmed death ligand 1.

**Table S5.** Descriptive statistics for septic shock patients (N = 30).

|  | Lymphocyte D1 (%) | Lymphocyte D5 (%) | CD4+  D1 (%) | CD4+  D5 (%) | CD8+  D1 (%) | CD8+  D5 (%) | NKT CD3+  D1 (%) | NKT CD3+  D5 (%) |
| --- | --- | --- | --- | --- | --- | --- | --- | --- |
| Number of values | 30 | 17 | 30 | 17 | 30 | 17 | 29 | 17 |
|  |  |  |  |  |  |  |  |  |
| Minimum | 0.02000 | 0.008000 | 0.2380 | 0.4400 | 0.04200 | 0.04800 | 0.005000 | 0.000 |
| 25% Percentile | 0.03175 | 0.03100 | 0.5863 | 0.5970 | 0.1975 | 0.2230 | 0.02350 | 0.01250 |
| Median | 0.05025 | 0.06400 | 0.6795 | 0.6780 | 0.2410 | 0.2600 | 0.05500 | 0.02900 |
| 75% Percentile | 0.08833 | 0.1084 | 0.7435 | 0.7510 | 0.3235 | 0.3130 | 0.1065 | 0.1000 |
| Maximum | 0.1690 | 0.3070 | 0.9300 | 0.9440 | 0.5040 | 0.4940 | 0.2050 | 0.1770 |
| Range | 0.1490 | 0.2990 | 0.6920 | 0.5040 | 0.4620 | 0.4460 | 0.2000 | 0.1770 |
|  |  |  |  |  |  |  |  |  |
| Mean | 0.06174 | 0.08446 | 0.6550 | 0.6774 | 0.2629 | 0.2748 | 0.06641 | 0.06047 |
| Std. Deviation | 0.03711 | 0.07500 | 0.1481 | 0.1262 | 0.1059 | 0.09956 | 0.04990 | 0.05508 |
| Std. Error of Mean | 0.006775 | 0.01819 | 0.02704 | 0.03062 | 0.01933 | 0.02415 | 0.009265 | 0.01336 |
|  |  |  |  |  |  |  |  |  |
| Coefficient of variation | 60.11% | 88.80% | 22.61% | 18.64% | 40.28% | 36.23% | 75.13% | 91.08% |
|  |  |  |  |  |  |  |  |  |
| Skewness | 1.236 | 1.885 | -0.8777 | -0.03258 | 0.5444 | -0.02652 | 0.9797 | 0.6454 |
| Kurtosis | 1.121 | 4.097 | 1.290 | 0.3354 | 0.2705 | 1.488 | 0.5618 | -0.7061 |
|  |  |  |  |  |  |  |  |  |
|  | CD19+  D1 (%) | CD19+  D5 (%) | PD-1  D1 (ng/ml) | PD-1  D5 (ng/ml) | PD-L1  D1 (ng/ml) | PD-L1  D5 (ng/ml) | IL-7  D1 (pg/ml) | IL-7  D5 (pg/ml) |
| Number of values | 29 | 17 | 29 | 17 | 27 | 16 | 29 | 15 |
|  |  |  |  |  |  |  |  |  |
| Minimum | 0.01000 | 0.001000 | 0.02700 | 0.01200 | 0.9890 | 1.128 | 0.1620 | 0.6890 |
| 25% Percentile | 0.08050 | 0.05350 | 0.09600 | 0.1170 | 5.127 | 3.557 | 3.032 | 1.687 |
| Median | 0.1730 | 0.1550 | 0.2160 | 0.2070 | 6.967 | 4.998 | 5.415 | 3.459 |
| 75% Percentile | 0.2690 | 0.2905 | 0.3475 | 0.3330 | 9.257 | 7.502 | 8.382 | 5.739 |
| Maximum | 0.5150 | 0.5200 | 1.256 | 1.055 | 24.58 | 12.30 | 37.43 | 15.33 |
| Range | 0.5050 | 0.5190 | 1.229 | 1.043 | 23.59 | 11.18 | 37.27 | 14.64 |
|  |  |  |  |  |  |  |  |  |
| Mean | 0.1918 | 0.1842 | 0.2934 | 0.2664 | 7.437 | 5.510 | 6.941 | 4.665 |
| Std. Deviation | 0.1281 | 0.1475 | 0.2897 | 0.2410 | 4.630 | 3.202 | 7.263 | 4.096 |
| Std. Error of Mean | 0.02379 | 0.03576 | 0.05379 | 0.05845 | 0.8911 | 0.8005 | 1.349 | 1.058 |
|  |  |  |  |  |  |  |  |  |
| Coefficient of variation | 66.81% | 80.06% | 98.74% | 90.46% | 62.25% | 58.11% | 104.6% | 87.80% |
|  |  |  |  |  |  |  |  |  |
| Skewness | 0.8223 | 0.8056 | 1.914 | 2.375 | 1.909 | 0.4911 | 2.973 | 1.627 |
| Kurtosis | 0.06623 | -0.05291 | 3.817 | 7.134 | 6.409 | -0.2081 | 11.17 | 2.233 |

Legend: CD: cluster of differentiation, CD4+: T helper CD4+ lymphocytes, CD8+: T cytotoxic CD8+ lymphocytes, CD19+: B CD19+ lymphocytes, D1: day 1, D5: day 5, IL-7: interleukin-7, NKT CD3+: natural killer T lymphocytes, PD-1: Programmed cell death protein 1, PD-L1: Programmed death ligand 1.

**Table S6**. Comparison of the studied parameters between sepsis and septic shock patients on day 1 and day 5 (median value and IQR).

| Parameter | Sepsis | | *p*^a^ value | Septic shock | | *p*^a^ value |
| --- | --- | --- | --- | --- | --- | --- |
|  | Day 1 | Day 5 |  | Day 1 | Day 5 |  |
| Th cells (CD4+), % | 0.6040 (0.2245) | 0.6500 (0.192) | 0.7493 | 0.6795 (0.1572) | 0.6780 (0.154) | 0.2683 |
| Tc cells (CD8+), % | 0.3450 (0.2185) | 0.3000 (0.176) | 0.8683 | 0.2410 (0.126) | 0.2600 (0.09) | 0.5477 |
| NKT (CD3+), % | 0.06650 (0.0868) | 0.06450 (0.08625) | 0.6214 | 0.05500 (0.083) | 0.02900 (0.0875) | 0.2582 |
| B cells (CD19+), % | 0.1200 (0.132) | 0.08700 (0.099) | 0.4624 | 0.1730 (0.1885) | 0.1550 (0.237) | 0.3964 |
| PD-1, ng/ml | 0.1600 (0.1255) | 0.1435 (0.12075) | 0.3393 | 0.2160 (0.2785) | 0.2070 (0.216) | >0.9999 |
| PD-L1, ng/ml | 5.523 (4.95) | 5.825 (3.9) | 0.8613 | 6.967 (4.13) | 4.998 (3.945) | **0.0353** |
| IL-7, pg/ml | 3.459 (4.204) | 2.382 (2.189) | 0.1557 | 5.415 (5.35) | 3.459 (4.052) | 0.0946 |

Legend: ^a^Wilcoxon test. Bold type indicates significance. B cells: B CD19+ lymphocytes, CD: cluster of differentiation, IL-7: interleukin-7, NKT CD3+: natural killer T CD3+ lymphocytes, PD-1: programmed cell death protein 1, PD-L1: programmed death ligand 1, Tc cells: T cytotoxic CD8+ lymphocytes, Th cells: T helper CD4+ lymphocytes.

**Table S7**. Correlations for the lot of sepsis patients on day 1 and day 5.

|  |  | Tc cells (CD8+), % | NKT (CD3+), % | B cells (CD19+), % | PD-1, ng/ml | PD-L1, ng/ml | IL-7, pg/ml |
| --- | --- | --- | --- | --- | --- | --- | --- |
| Th cells (CD4+), % | Day 1 | r = -0.8654  (-0.9188 to -0.7809)  ***p*^b^ <0.0001** | r = -0.5418  (-0.7110 to -0.3132)  ***p*^a^ <0.0001** | r = 0.1048  (-0.1729 to 0.3670)  *p*^a^ = 0.4466 | r = 0.1386  (-0.1449 to 0.4010)  *p*^a^ = 0.3224 | r = 0.1374  (-0.1496 to 0.4030)  *p*^b^ = 0.3466 | r = 0.03404  (-0.2435 to 0.3064)  *p*^a^ = 0.8069 |
|  | Day 5 | r = -0.9776  (-0.9887 to -0.9557)  ***p*^b^ <0.0001** | r = -0.5652  (-0.7628 to -0.2710)  ***p*^a^ = 0.0005** | r = 0.3342  (-0.02090 to 0.6144)  *p*^a^ = 0.0573 | r = -0.2788  (-0.5881 to 0.1016)  *p*^a^ = 0.1357 | r = -0.001538  (-0.4068 to 0.4042)  *p*^a^ = 0.9942 | r = -0.1704  (-0.5138 to 0.2200)  *p*^a^ = 0.3767 |
| Tc cells (CD8+), % | Day 1 |  | r = 0.6355  (0.4367 to 0.7752)  ***p*^a^ <0.0001** | r = -0.2397  (-0.4810 to 0.03537)  *p*^a^ = 0.0780 | r = -0.1885  (-0.4431 to 0.09433)  *p*^a^ = 0.1765 | r = -0.2057  (-0.4603 to 0.08011)  *p*^b^ = 0.1562 | r = -0.07636  (-0.3444 to 0.2032)  *p*^a^ = 0.5831 |
|  | Day 5 |  | r = 0.5217  (0.2130 to 0.7358)  ***p*^a^ = 0.0016** | r = -0.2965  (-0.5877 to 0.06267)  *p*^a^ = 0.0938 | r = 0.2487  (-0.1336 to 0.5665)  *p*^a^ = 0.1852 | r = 0.1078  (-0.3113 to 0.4918)  *p*^a^ = 0.6081 | r = 0.1870  (-0.2036 to 0.5263)  *p*^a^ = 0.3314 |
| NKT (CD3+), % | Day 1 |  |  | r = -0.3074  (-0.5412 to -0.02935)  ***p*^a^ = 0.0267** | r = -0.2421  (-0.4917 to 0.04418)  *p*^a^ = 0.0869 | r = -0.06482  (-0.3502 to 0.2316)  *p*^a^ = 0.6616 | r = -0.1290  (-0.3978 to 0.1601)  *p*^a^ = 0.3668 |
|  | Day 5 |  |  | r = -0.2532  (-0.5605 to 0.1154)  *p*^a^ = 0.1620 | r = -0.2033  (-0.5384 to 0.1873)  *p*^a^ = 0.2902 | r = -0.2775  (-0.6202 to 0.1541)  *p*^a^ = 0.1892 | r = 0.005203  (-0.3786 to 0.3874)  *p*^a^ = 0.9790 |
| B cells (CD19+), % | Day 1 |  |  |  | r = 0.2205  (-0.06702 to 0.4741)  *p*^a^ = 0.1201 | r = -0.02095  (-0.3111 to 0.2728)  *p*^a^ = 0.8876 | r = 0.2358  (-0.04501 to 0.4821)  *p*^a^ = 0.0892 |
|  | Day 5 |  |  |  | r = -0.06000  (-0.4267 to 0.3236)  *p*^a^ = 0.7572 | r = -0.1586  (-0.5450 to 0.2833)  *p*^a^ = 0.4697 | r = -0.04825  (-0.4302 to 0.3484)  *p*^a^ = 0.8111 |
| PD-1, ng/ml | Day 1 |  |  |  |  | r = 0.002837  (-0.2958 to 0.3010)  *p*^a^ = 0.9851 | r = 0.1429  (-0.1493 to 0.4122)  *p*^a^ = 0.3221 |
|  | Day 5 |  |  |  |  | r = -0.06957  (-0.4700 to 0.3546)  *p*^a^ = 0.7467 | r = 0.2644  (-0.1320 to 0.5879)  *p*^a^ = 0.1739 |
| PD-L1, ng/ml | Day 1 |  |  |  |  |  | r = -0.01214  (-0.3062 to 0.2840)  *p*^a^ = 0.9354 |
|  | Day 5 |  |  |  |  |  | r = 0.2504  (-0.1824 to 0.6020)  *p*^a^ = 0.2379 |

Legend: ^a^Spearman test, ^b^Pearson test. Bold type indicates significance. B cells: B CD19+ lymphocytes, CD: cluster of differentiation, IL-7: interleukin-7, NKT CD3+: natural killer T CD3+ lymphocytes, PD-1: programmed cell death protein 1, PD-L1: programmed death ligand 1, Tc cells: T cytotoxic CD8+ lymphocytes, Th cells: T helper CD4+ lymphocytes.

,

**Table S8**. Correlations for the lot of septic shock patients on day 1 and day 5.

|  |  | Tc cells (CD8+), % | NKT (CD3+), % | B cells (CD19+), % | PD-1, ng/ml | PD-L1, ng/ml | IL-7, pg/ml |
| --- | --- | --- | --- | --- | --- | --- | --- |
| Th cells (CD4+), % | Day 1 | r = -0.4830  (-0.7183 to -0.1486)  ***p*^b^ = 0.0069** | r = -0.7446  (-0.8756 to -0.5117)  ***p*^a^ <0.0001** | r = 0.1272  (-0.2616 to 0.4805)  *p*^a^ = 0.5109 | r = 0.2340  (-0.1561 to 0.5609)  *p*^a^ = 0.2219 | r = 0.06571  (-0.3224 to 0.4349)  *p*^a^ = 0.7447 | r = 0.3342  (-0.04816 to 0.6311)  *p*^a^ = 0.0764 |
|  | Day 5 | r = -0.9042  (-0.9653 to -0.7491)  ***p*^b^ <0.0001** | r = -0.5611  (-0.8255 to -0.09482)  ***p*^a^ = 0.0208** | r = 0.08795  (-0.4100 to 0.5455)  *p*^b^ = 0.7371 | r = -0.03431  (-0.5180 to 0.4660)  *p*^a^ = 0.8984 | r = -0.4075  (-0.7514 to 0.1105)  *p*^a^ = 0.1172 | r = -0.2143  (-0.6641 to 0.3495)  *p*^a^ = 0.4421 |
| Tc cells (CD8+), % | Day 1 |  | r = 0.3034  (-0.08225 to 0.6101)  *p*^a^ = 0.1096 | r = 0.2028  (-0.1878 to 0.5381)  *p*^a^ = 0.2913 | r = -0.3345  (-0.6313 to 0.04784)  *p*^a^ = 0.0761 | r = -0.04149  (-0.4150 to 0.3439)  *p*^a^ = 0.8372 | r = -0.1846  (-0.5244 to 0.2061)  *p*^a^ = 0.3379 |
|  | Day 5 |  | r = 0.2910  (-0.2352 to 0.6852)  *p*^a^ = 0.2553 | r = -0.1008  (-0.5546 to 0.3992)  *p*^b^ = 0.7002 | r = -0.02696  (-0.5126 to 0.4718)  *p*^a^ = 0.9209 | r = 0.4329  (-0.08000 to 0.7645)  *p*^b^ = 0.0940 | r = -0.03214  (-0.5474 to 0.5008)  *p*^a^ = 0.9132 |
| NKT (CD3+), % | Day 1 |  |  | r = -0.3174  (-0.6245 to 0.07470)  *p*^a^ = 0.0998 | r = -0.1497  (-0.5038 to 0.2475)  *p*^a^ = 0.4471 | r = 0.2596  (-0.1539 to 0.5957)  *p*^a^ = 0.2003 | r = -0.1346  (-0.4922 to 0.2620)  *p*^a^ = 0.4948 |
|  | Day 5 |  |  | r = -0.3131  (-0.6979 to 0.2121)  *p*^a^ = 0.2197 | r = 0.01596  (-0.4803 to 0.5045)  *p*^a^ = 0.9528 | r = -0.02653  (-0.5272 to 0.4878)  *p*^a^ = 0.9234 | r = 0.1594  (-0.3984 to 0.6311)  *p*^a^ = 0.5678 |
| B cells (CD19+), % | Day 1 |  |  |  | r = -0.1896  (-0.5339 to 0.2085)  *p*^a^ = 0.3338 | r = -0.3269  (-0.6412 to 0.08117)  *p*^a^ = 0.1030 | r = -0.1188  (-0.4800 to 0.2768)  *p*^a^ = 0.5471 |
|  | Day 5 |  |  |  | r = -0.3848  (-0.7375 to 0.1328)  *p*^a^ = 0.1281 | r = -0.05517  (-0.5362 to 0.4529)  *p*^a^ = 0.8392 | r = -0.4857  (-0.8051 to 0.05204)  *p*^a^ = 0.0688 |
| PD-1, ng/ml | Day 1 |  |  |  |  | r = 0.4566  (0.07218 to 0.7230)  ***p*^a^ = 0.0190** | r = 0.2940  (-0.1003 to 0.6085)  *p*^a^ = 0.1288 |
|  | Day 5 |  |  |  |  | r = 0.3088  (-0.2359 to 0.7059)  *p*^a^ = 0.2440 | r = 0.5750  (0.07231 to 0.8447)  ***p*^a^ = 0.0274** |
| PD-L1, ng/ml | Day 1 |  |  |  |  |  | r = 0.02154  (-0.3793 to 0.4156)  *p*^a^ = 0.9168 |
|  | Day 5 |  |  |  |  |  | r = 0.2393  (-0.3261 to 0.6786)  *p*^a^ = 0.3892 |

Legend: ^a^Spearman test, ^b^Pearson test. Bold type indicates significance. B cells: B CD19+ lymphocytes, CD: cluster of differentiation, IL-7: interleukin-7, NKT CD3+: natural killer T CD3+ lymphocytes, PD-1: programmed cell death protein 1, PD-L1: programmed death ligand 1, Tc cells: T cytotoxic CD8+ lymphocytes, Th cells: T helper CD4+ lymphocytes.

**Table S9**. Descriptive statistics for survivor patients (N = 24).

|  | Lymphocyte D1 (%) | Lymphocyte  D5 (%) | CD4+  D1 (%) | CD4+  D5 (%) | CD8+  D1 (%) | CD8+  D5 (%) | NKT CD3+  D1 (%) | NKT CD3+  D5 (%) |
| --- | --- | --- | --- | --- | --- | --- | --- | --- |
| Number of values | 24 | 16 | 24 | 18 | 24 | 18 | 22 | 17 |
|  |  |  |  |  |  |  |  |  |
| Minimum | 0.02900 | 0.02500 | 0.4160 | 0.4340 | 0.1441 | 0.1450 | 0.000 | 0.006000 |
| 25% Percentile | 0.04123 | 0.06850 | 0.5618 | 0.5285 | 0.2065 | 0.2265 | 0.03050 | 0.02150 |
| Median | 0.06520 | 0.1160 | 0.6635 | 0.6535 | 0.2800 | 0.3060 | 0.05500 | 0.08800 |
| 75% Percentile | 0.09925 | 0.1815 | 0.7208 | 0.7430 | 0.3908 | 0.4230 | 0.09900 | 0.1230 |
| Maximum | 0.1690 | 0.3070 | 0.8300 | 0.8360 | 0.4710 | 0.5610 | 0.1760 | 0.2940 |
| Range | 0.1400 | 0.2820 | 0.4140 | 0.4020 | 0.3269 | 0.4160 | 0.1760 | 0.2880 |
|  |  |  |  |  |  |  |  |  |
| Mean | 0.07612 | 0.1266 | 0.6509 | 0.6365 | 0.3005 | 0.3263 | 0.06686 | 0.09041 |
| Std. Deviation | 0.04119 | 0.07542 | 0.1105 | 0.1312 | 0.1006 | 0.1276 | 0.04736 | 0.07270 |
| Std. Error of Mean | 0.008409 | 0.01886 | 0.02256 | 0.03092 | 0.02053 | 0.03008 | 0.01010 | 0.01763 |
|  |  |  |  |  |  |  |  |  |
| Coefficient of variation | 54.12% | 59.60% | 16.98% | 20.61% | 33.47% | 39.11% | 70.83% | 80.41% |
|  |  |  |  |  |  |  |  |  |
| Skewness | 0.9149 | 0.8203 | -0.2240 | -0.2087 | 0.1214 | 0.4695 | 0.6784 | 1.288 |
| Kurtosis | -0.1395 | 0.6945 | -0.5960 | -0.9308 | -1.424 | -0.8223 | -0.2097 | 2.686 |
|  |  |  |  |  |  |  |  |  |
|  | CD19+  D1 (%) | CD19+  D5 (%) | PD-1  D1 (ng/ml) | PD-1  D5 (ng/ml) | PD-L1  D1 (ng/ml) | PD-L1  D5 (ng/ml) | IL-7  D1 (pg/ml) | IL-7  D5 (pg/ml) |
| Number of values | 24 | 16 | 24 | 16 | 20 | 15 | 23 | 16 |
|  |  |  |  |  |  |  |  |  |
| Minimum | 0.01000 | 0.02500 | 0.04400 | 0.01200 | 0.9210 | 0.7700 | 1.293 | 0.6380 |
| 25% Percentile | 0.07775 | 0.05950 | 0.09325 | 0.07750 | 3.585 | 4.156 | 2.199 | 2.175 |
| Median | 0.1170 | 0.09350 | 0.1750 | 0.1880 | 5.170 | 5.249 | 3.921 | 3.227 |
| 75% Percentile | 0.1620 | 0.1573 | 0.2373 | 0.3680 | 7.297 | 6.808 | 6.545 | 5.212 |
| Maximum | 0.5680 | 0.2810 | 0.9640 | 0.5510 | 18.65 | 26.06 | 37.43 | 10.47 |
| Range | 0.5580 | 0.2560 | 0.9200 | 0.5390 | 17.73 | 25.29 | 36.14 | 9.831 |
|  |  |  |  |  |  |  |  |  |
| Mean | 0.1441 | 0.1123 | 0.2328 | 0.2315 | 5.753 | 6.505 | 5.790 | 4.088 |
| Std. Deviation | 0.1177 | 0.07075 | 0.2175 | 0.1787 | 3.845 | 5.672 | 7.320 | 3.205 |
| Std. Error of Mean | 0.02403 | 0.01769 | 0.04440 | 0.04467 | 0.8597 | 1.464 | 1.526 | 0.8013 |
|  |  |  |  |  |  |  |  |  |
| Coefficient of variation | 81.69% | 63.03% | 93.42% | 77.19% | 66.84% | 87.20% | 126.4% | 78.41% |
|  |  |  |  |  |  |  |  |  |
| Skewness | 2.334 | 1.015 | 2.169 | 0.6842 | 2.045 | 3.259 | 3.973 | 1.246 |
| Kurtosis | 6.946 | 0.6629 | 5.069 | -0.7186 | 6.246 | 11.90 | 17.44 | 0.3023 |

Legend: CD: cluster of differentiation, CD4+: T helper CD4+ lymphocytes, CD8+: T cytotoxic CD8+ lymphocytes, CD19+: B CD19+ lymphocytes, D1: day 1, D5: day 5, IL-7: interleukin-7, NKT CD3+: natural killer T lymphocytes, PD-1: Programmed cell death protein 1, PD-L1: Programmed death ligand 1.

**Table S10**. Descriptive statistics for non-survivor patients (N = 63).

|  | Lymphocyte  D1 (%) | Lymphocyte  D5 (%) | CD4+  D1 (%) | CD4+  D5 (%) | CD8+  D1 (%) | CD8+  D5 (%) | NKT CD3+  D1 (%) | NKT CD3+  D5 (%) |
| --- | --- | --- | --- | --- | --- | --- | --- | --- |
| Number of values | 63 | 36 | 63 | 34 | 63 | 34 | 61 | 34 |
|  |  |  |  |  |  |  |  |  |
| Minimum | 0.01800 | 0.008000 | 0.2380 | 0.2800 | 0.04200 | 0.04800 | 0.000 | 0.000 |
| 25% Percentile | 0.03500 | 0.03825 | 0.5230 | 0.5625 | 0.2040 | 0.2368 | 0.02450 | 0.02225 |
| Median | 0.05700 | 0.06350 | 0.6220 | 0.6790 | 0.2930 | 0.3000 | 0.06000 | 0.04900 |
| 75% Percentile | 0.09500 | 0.09200 | 0.7500 | 0.7468 | 0.4280 | 0.3648 | 0.1155 | 0.09650 |
| Maximum | 0.5580 | 0.3170 | 0.9300 | 0.9440 | 0.6880 | 0.5670 | 0.5380 | 0.3200 |
| Range | 0.5400 | 0.3090 | 0.6920 | 0.6640 | 0.6460 | 0.5190 | 0.5380 | 0.3200 |
|  |  |  |  |  |  |  |  |  |
| Mean | 0.08036 | 0.07939 | 0.6284 | 0.6633 | 0.3094 | 0.2974 | 0.08244 | 0.06818 |
| Std. Deviation | 0.08270 | 0.06174 | 0.1587 | 0.1372 | 0.1392 | 0.1162 | 0.08656 | 0.06555 |
| Std. Error of Mean | 0.01042 | 0.01029 | 0.01999 | 0.02353 | 0.01754 | 0.01993 | 0.01108 | 0.01124 |
|  |  |  |  |  |  |  |  |  |
| Coefficient of variation | 102.9% | 77.77% | 25.26% | 20.68% | 44.99% | 39.09% | 105.0% | 96.15% |
|  |  |  |  |  |  |  |  |  |
| Skewness | 3.998 | 2.028 | -0.2709 | -0.4637 | 0.3637 | 0.2598 | 2.969 | 2.004 |
| Kurtosis | 19.52 | 5.296 | -0.3186 | 0.9391 | -0.3905 | 0.5428 | 12.74 | 5.566 |
|  |  |  |  |  |  |  |  |  |
|  | CD19+  D1 (%) | CD19+  D5 (%) | PD-1  D1 (ng/ml) | PD-1  D5 (ng/ml) | PD-L1  D1 (ng/ml) | PD-L1  D5 (ng/ml) | IL-7  D1 (pg/ml) | IL-7  D5 (pg/ml) |
| Number of values | 60 | 34 | 58 | 33 | 56 | 28 | 60 | 30 |
|  |  |  |  |  |  |  |  |  |
| Minimum | 0.01000 | 0.001000 | 0.01200 | 0.01300 | 0.3830 | 1.128 | 0.1620 | 0.008000 |
| 25% Percentile | 0.08100 | 0.04325 | 0.1030 | 0.09900 | 3.338 | 3.720 | 1.426 | 1.361 |
| Median | 0.1510 | 0.1090 | 0.1855 | 0.1570 | 6.964 | 5.814 | 4.381 | 2.542 |
| 75% Percentile | 0.2465 | 0.2678 | 0.2680 | 0.2270 | 8.854 | 8.948 | 7.260 | 4.047 |
| Maximum | 0.7300 | 0.5200 | 1.256 | 1.055 | 24.58 | 12.30 | 20.90 | 15.33 |
| Range | 0.7200 | 0.5190 | 1.244 | 1.042 | 24.19 | 11.18 | 20.74 | 15.33 |
|  |  |  |  |  |  |  |  |  |
| Mean | 0.1822 | 0.1592 | 0.2370 | 0.2074 | 6.562 | 6.157 | 4.818 | 3.300 |
| Std. Deviation | 0.1444 | 0.1410 | 0.2120 | 0.2020 | 4.151 | 3.240 | 3.908 | 3.228 |
| Std. Error of Mean | 0.01864 | 0.02418 | 0.02783 | 0.03517 | 0.5547 | 0.6124 | 0.5045 | 0.5893 |
|  |  |  |  |  |  |  |  |  |
| Coefficient of variation | 79.28% | 88.55% | 89.45% | 97.43% | 63.25% | 52.63% | 81.11% | 97.81% |
|  |  |  |  |  |  |  |  |  |
| Skewness | 1.476 | 1.100 | 2.621 | 2.776 | 1.307 | -0.003926 | 1.464 | 2.362 |
| Kurtosis | 2.670 | 0.2709 | 9.037 | 9.433 | 5.075 | -0.9375 | 3.619 | 6.652 |

Legend: CD: cluster of differentiation, CD4+: T helper CD4+ lymphocytes, CD8+: T cytotoxic CD8+ lymphocytes, CD19+: B CD19+ lymphocytes, D1: day 1, D5: day 5, IL-7: interleukin-7, NKT CD3+: natural killer T lymphocytes, PD-1: Programmed cell death protein 1, PD-L1: Programmed death ligand 1.

**Table S11**. Comparison of the studied parameters between survivors and non-survivor patients on day 1 and day 5 (median value and IQR).

| Parameter | Survivors | | *p*^a^ value | Non-survivors | | *p*^a^ value |
| --- | --- | --- | --- | --- | --- | --- |
|  | Day 1 | Day 5 |  | Day 1 | Day 5 |  |
| Th cells (CD4+), % | 0.6635 (0.159) | 0.6535 (0.2145) | 0.4233 | 0.6220 (0.227) | 0.6790 (0.1843) | 0.9764 |
| Tc cells (CD8+), % | 0.2800 (0.1843) | 0.3060 (0.1965) | 0.2601 | 0.2930 (0.224) | 0.3000 (0.128) | 0.9093 |
| NKT (CD3+), % | 0.05500 (0.0595) | 0.08800 (0.1015) | **0.0076** | 0.06000 (0.091) | 0.04900 (0.07425) | 0.4346 |
| B cells (CD19+), % | 0.1170 (0.08425) | 0.09350 (0.0978) | 0.4884 | 0.1510 (0.1655) | 0.1090 (0.2245) | 0.4238 |
| PD-1, ng/ml | 0.1750 (0.14405) | 0.1880 (0.2905) | 0.8603 | 0.1855 (0.165) | 0.1570 (0.128) | 0.2929 |
| PD-L1, ng/ml | 5.170 (3.712) | 5.249 (2.652) | 0.5830 | 6.964 (5.516) | 5.814 (5.228) | 0.3242 |
| IL-7, pg/ml | 3.921 (4.346) | 3.227 (3.037) | 0.9780 | 4.381 (5.834) | 2.542 (2.686) | **0.0069** |

Legend: ^a^Wilcoxon test. Bold type indicates significance. B cells: B CD19+ lymphocytes, CD: cluster of differentiation, IL-7: interleukin-7, NKT CD3+: natural killer T CD3+ lymphocytes, PD-1: programmed cell death protein 1, PD-L1: programmed death ligand 1, Tc cells: T cytotoxic CD8+ lymphocytes, Th cells: T helper CD4+ lymphocytes.

**Table S12**. Correlations for the lot of survivor patients on day 1 and day 5.

|  |  | Tc cells (CD8+), % | NKT, % | B cells (CD19+), % | PD-1, ng/ml | PD-L1, ng/ml | IL-7, pg/ml |
| --- | --- | --- | --- | --- | --- | --- | --- |
| Th cells (CD4+), % | Day 1 | r = -0.9368  (-0.9726 to -0.8575)  ***p*^b^ <0.0001** | r = -0.4108  (-0.7095 to 0.01305)  *p*^b^ = 0.0575 | r = 0.3740  (-0.04725 to 0.6823)  *p*^a^ = 0.0718 | r = -0.2344  (-0.5910 to 0.1988)  *p*^a^ = 0.2703 | r = 0.1030  (-0.3679 to 0.5319)  *p*^a^ = 0.6655 | r = 0.2753  (-0.1670 to 0.6254)  *p*^a^ = 0.2035 |
|  | Day 5 | r = -0.9788  (-0.9922 to -0.9426)  ***p*^b^ <0.0001** | r = -0.6998  (-0.8833 to -0.3302)  ***p*^b^ = 0.0018** | r = 0.6929  (0.3004 to 0.8847)  ***p*^b^ = 0.0029** | r = 0.05296  (-0.4547 to 0.5346)  *p*^b^ = 0.8456 | r = -0.06786  (-0.5720 to 0.4735)  *p*^a^ = 0.8124 | r = 0.02941  (-0.4856 to 0.5292)  *p*^a^ = 0.9171 |
| Tc cells (CD8+), % | Day 1 |  | r = 0.5205  (0.1267 to 0.7726)  ***p*^b^ = 0.0130** | r = -0.4517  (-0.7293 to -0.04645)  ***p*^a^ = 0.0267** | r = 0.1723  (-0.2602 to 0.5472)  *p*^a^ = 0.4207 | r = -0.07446  (-0.5110 to 0.3926)  *p*^a^ = 0.7550 | r = -0.2330  (-0.5971 to 0.2107)  *p*^a^ = 0.2847 |
|  | Day 5 |  | r = 0.6379  (0.2269 to 0.8561)  ***p*^b^ = 0.0059** | r = -0.6794  (-0.8791 to -0.2770)  ***p*^b^ = 0.0038** | r = -0.01212  (-0.5048 to 0.4865)  *p*^b^ = 0.9645 | r = 0.1893  (-0.3722 to 0.6493)  *p*^a^ = 0.4983 | r = -0.1176  (-0.5901 to 0.4149)  *p*^a^ = 0.6645 |
| NKT, % | Day 1 |  |  | r = -0.4864  (-0.7592 to -0.06832)  ***p*^a^ = 0.0217** | r = 0.1725  (-0.2809 to 0.5630)  *p*^a^ = 0.4427 | r = -0.07476  (-0.5222 to 0.4050)  *p*^a^ = 0.7610 | r = -0.3839  (-0.7065 to 0.07092)  *p*^a^ = 0.0858 |
|  | Day 5 |  |  | r = -0.4824  (-0.7976 to 0.03964)  *p*^b^ = 0.0686 | r = -0.4909  (-0.8016 to 0.02859)  *p*^b^ = 0.0632 | r = -0.2992  (-0.7245 to 0.2911)  *p*^a^ = 0.2965 | r = -0.2181  (-0.6663 to 0.3460)  *p*^a^ = 0.4318 |
| B cells (CD19+), % | Day 1 |  |  |  | r = -0.2339  (-0.5907 to 0.1993)  *p*^a^ = 0.2713 | r = -0.07068  (-0.5081 to 0.3958)  *p*^a^ = 0.7672 | r = 0.07660  (-0.3579 to 0.4838)  *p*^a^ = 0.7283 |
|  | Day 5 |  |  |  | r = -0.1500  (-0.6150 to 0.3924)  *p*^b^ = 0.5937 | r = -0.1978  (-0.6850 to 0.4117)  *p*^a^ = 0.5171 | r = 0.09011  (-0.4762 to 0.6036)  *p*^a^ = 0.7616 |
| PD-1, ng/ml | Day 1 |  |  |  |  | r = -0.1835  (-0.5882 to 0.2948)  *p*^a^ = 0.4388 | r = 0.1977  (-0.2458 to 0.5727)  *p*^a^ = 0.3659 |
|  | Day 5 |  |  |  |  | r = 0.05934  (-0.4998 to 0.5836)  *p*^a^ = 0.8438 | r = 0.2484  (-0.3406 to 0.6973)  *p*^a^ = 0.3911 |
| PD-L1, ng/ml | Day 1 |  |  |  |  |  | r = 0.1596  (-0.3306 to 0.5820)  *p*^a^ = 0.5138 |
|  | Day 5 |  |  |  |  |  | r = 0.06786  (-0.4735 to 0.5720)  *p*^a^ = 0.8124 |

Legend: ^a^Spearman test, ^b^Pearson test. Bold type indicates significance. B cells: B CD19+ lymphocytes, CD: cluster of differentiation, IL-7: interleukin-7, NKT CD3+: natural killer T CD3+ lymphocytes, PD-1: programmed cell death protein 1, PD-L1: programmed death ligand 1, Tc cells: T cytotoxic CD8+ lymphocytes, Th cells: T helper CD4+ lymphocytes.

**Table S13**. Correlations for the lot of non-survivor patients on day 1 and day 5.

|  |  | Tc cells (CD8+), % | NKT, % | B cells (CD19+), % | PD-1, ng/ml | PD-L1, ng/ml | IL-7, pg/ml |
| --- | --- | --- | --- | --- | --- | --- | --- |
| Th cells (CD4+), % | Day 1 | r = -0.7114  (-0.8155 to -0.5629)  ***p*^b^ <0.0001** | r = -0.6415  (-0.7722 to -0.4588)  ***p*^a^ <0.0001** | r = 0.1206  (-0.1451 to 0.3700)  *p*^a^ = 0.3588 | r = 0.3463  (0.08886 to 0.5603)  ***p*^a^ = 0.0078** | r = 0.1843  (-0.08264 to 0.4265)  *p*^b^ = 0.1740 | r = 0.1172  (-0.1484 to 0.3671)  *p*^a^ = 0.3723 |
|  | Day 5 | r = -0.9461  (-0.9730 to -0.8939)  ***p*^b^ <0.0001** | r = -0.4962  (-0.7145 to -0.1899)  ***p*^b^ = 0.0028** | r = 0.1412  (-0.2168 to 0.4657)  *p*^a^ = 0.4257 | r = -0.4123  (-0.6749 to -0.05702)  ***p*^a^ = 0.0212** | r = -0.2116  (-0.5536 to 0.1915)  *p*^b^ = 0.2995 | r = -0.1133  (-0.4757 to 0.2819)  *p*^a^ = 0.5659 |
| Tc cells (CD8+), % | Day 1 |  | r = 0.5488  (0.3379 to 0.7072)  ***p*^a^ <0.0001** | r = -0.1183  (-0.3680 to 0.1474)  *p*^a^ = 0.3682 | r = -0.3873  (-0.5920 to -0.1357)  ***p*^a^ = 0.0027** | r = -0.2790  (-0.5049 to -0.01736)  ***p*^b^ = 0.0373** | r = -0.1029  (-0.3545 to 0.1625)  *p*^a^ = 0.4339 |
|  | Day 5 |  | r = 0.4641  (0.1493 to 0.6934)  ***p*^b^ = 0.0057** | r = -0.08319  (-0.4184 to 0.2720)  *p*^a^ = 0.6400 | r = 0.3414  (-0.02566 to 0.6273)  *p*^a^ = 0.0602 | r = 0.2440  (-0.1584 to 0.5768)  *p*^b^ = 0.2297 | r = 0.1375  (-0.2592 to 0.4944)  *p*^a^ = 0.4855 |
| NKT, % | Day 1 |  |  | r = -0.2894  (-0.5153 to -0.02578)  ***p*^a^ = 0.0276** | r = -0.3663  (-0.5775 to -0.1091)  ***p*^a^ = 0.0051** | r = 0.003428  (-0.2696 to 0.2759)  *p*^a^ = 0.9802 | r = -0.05624  (-0.3171 to 0.2125)  *p*^a^ = 0.6750 |
|  | Day 5 |  |  | r = -0.2814  (-0.5728 to 0.07306)  *p*^a^ = 0.1069 | r = 0.05467  (-0.3155 to 0.4104)  *p*^a^ = 0.7702 | r = -0.1061  (-0.4739 to 0.2934)  *p*^b^ = 0.6061 | r = 0.1150  (-0.2803 to 0.4770)  *p*^a^ = 0.5600 |
| B cells (CD19+), % | Day 1 |  |  |  | r = 0.1884  (-0.08889 to 0.4386)  *p*^a^ = 0.1683 | r = -0.1443  (-0.4035 to 0.1364)  *p*^a^ = 0.2979 | r = 0.1398  (-0.1307 to 0.3908)  *p*^a^ = 0.2954 |
|  | Day 5 |  |  |  | r = -0.1472  (-0.4851 to 0.2289)  *p*^a^ = 0.4294 | r = -0.02838  (-0.4212 to 0.3734)  *p*^a^ = 0.8906 | r = -0.1754  (-0.5233 to 0.2225)  *p*^a^ = 0.3719 |
| PD-1, ng/ml | Day 1 |  |  |  |  | r = 0.2528  (-0.02984 to 0.4980)  *p*^a^ = 0.0706 | r = 0.2317  (-0.04387 to 0.4744)  *p*^a^ = 0.0888 |
|  | Day 5 |  |  |  |  | r = 0.03316  (-0.3693 to 0.4251)  *p*^a^ = 0.8722 | r = 0.3774  (0.001264 to 0.6600)  ***p*^a^ = 0.0436** |
| PD-L1, ng/ml | Day 1 |  |  |  |  |  | r = 0.008348  (-0.2675 to 0.2830)  *p*^a^ = 0.9522 |
|  | Day 5 |  |  |  |  |  | r = 0.2304  (-0.2028 to 0.5883)  *p*^a^ = 0.2787 |

Legend: ^a^Spearman test, ^b^Pearson test. Bold type indicates significance. B cells: B CD19+ lymphocytes, CD: cluster of differentiation, IL-7: interleukin-7, NKT CD3+: natural killer T CD3+ lymphocytes, PD-1: programmed cell death protein 1, PD-L1: programmed death ligand 1, Tc cells: T cytotoxic CD8+ lymphocytes, Th cells: T helper CD4+ lymphocytes.
